# In Vitro Spectroscopy-Based Profiling of Urothelial Carcinoma: A Fourier Transform Infrared and Raman Imaging Study

**DOI:** 10.3390/cancers13010123

**Published:** 2021-01-02

**Authors:** Monika Kujdowicz, Wojciech Placha, Brygida Mech, Karolina Chrabaszcz, Krzysztof Okoń, Kamilla Malek

**Affiliations:** 1 Department of Pathomorphology, Faculty of Medicine, Jagiellonian University Medical College, Grzegorzecka 16, 31-531 Krakow, Poland; monika.kujdowicz@uj.edu.pl (M.K.); k.okon@uj.edu.pl (K.O.); 2 Faculty of Chemistry, Jagiellonian University in Krakow, Gronostajowa 2, 30-387 Krakow, Poland; brygida.mech@student.uj.edu.pl (B.M.); karolina.chrabaszcz@doctoral.uj.edu.pl (K.C.); 3 Chair of Medical Biochemistry, Faculty of Medicine, Jagiellonian University Medical College, Kopernika 7, 31-034 Krakow, Poland; wojciech.placha@uj.edu.pl

**Keywords:** bladder cancer, infrared spectroscopy, Raman spectroscopy, molecular differentiation, cell lines

## Abstract

**Simple Summary:**

The mortality and recurrence associated with urothelial carcinoma are high. High heterogeneity makes it hard to detect with currently available methods such as cytology and histology. We propose here vibrational spectroscopic imaging as an additional diagnostic tool for the classification of bladder cancer. Our study revealed that chemism-induced spectroscopic features of the cancer cells of various stages and invasiveness were specifically detected.

**Abstract:**

Markers of bladder cancer cells remain elusive, which is a major cause of the low recognition of this malignant neoplasm and its recurrence. This implies an urgent need for additional diagnostic tools which are based on the identification of the chemism of bladder cancer. In this study, we employed label-free techniques of molecular imaging—Fourier Transform Infrared and Raman spectroscopic imaging—to investigate bladder cancer cell lines of various invasiveness (T24a, T24p, HT-1376, and J82). The urothelial HCV-29 cell line was the healthy control. Specific biomolecules discriminated spatial distribution of the nucleus and cytoplasm and indicated the presence of lipid bodies and graininess in some cell lines. The most prominent discriminators are the total content of lipids and sugar moieties as well as the presence of glycogen and other carbohydrates, un/saturated lipids, cytochromes, and a level of S-S bridges in proteins. The combination of the obtained hyperspectral database and chemometric methods showed a clear differentiation of each cell line at the level of the nuclei and cytoplasm and pointed out spectral signals which differentiated bladder cancer cells. Registered spectral markers correlated with biochemical composition changes can be associated with pathogenesis and potentially used for the diagnosis of bladder cancer and response to experimental therapies.

## 1. Introduction

Bladder cancer (BC) is one of the most common malignant neoplasms worldwide. The mortality rate and recurrence associated with it are both high. The transitional epithelium (urothelium) lines the entire urinary tract—renal pelvis, ureters, bladder and urethra. In the course of neoplasia, the urothelial cells differentiate in various ways and present multiple genetic alternations. Although histology is highly effective, low-grade cancer cells may not be distinguished from reactive changes using cytology, which is commonly used for screening and follow-up of patients. The above facts imply an urgent need for additional diagnostic tools and one that is proposed in our work is vibrational spectroscopy.

The pathogenic approach for a classification of urothelial bladder cancer (BC) relies on the distinction of the initial process between two pathways—luminal papillary and basal non-papillary [1]. The luminal changes include changes in epigenome and controlling its genes (i.e., mutations in STAB—encoding stabilin; HRAS—encoding GTPase HRas, also known as transforming protein p21) that in turn induce higher methylation of DNA and lower transcription (and RNA content) and translation. Subsequently, in response to these changes, cells counteract by, i.e., telomerase reverse transcriptase (TERT) mutation, the production of epidermal growth factor receptors (EGFR; the effect of fibroblast growth factor 3 (FGF3) gene mutation) and acceleration of the cell cycle (retinoblastoma gene (RB1) and TP53 mutations). Afterwards, the cells start to infiltrate the basal lamina and further tissues in a proteinase-dependent manner [2]. The basal pathway is shorter from the start of the invasion and metastasizes. Molecular subtyping of BC is a promising approach to predict clinical outcomes [3]. The other aspect is the applicable therapy (molecular and clinicopathological subtypes). This approach divides bladder cancer into five groups (TP53/cell cycle, chromatin-modifying, DNA damage and repair of related genes, PI3K/MAPK [phosphatidylinositol 3-kinase/ mitogen-activated protein kinase], genomic alternations and immune system response markers) [4,5]. Currently, the clinical standard in the early stages of BC entails treatment with surgical procedures (transurethral excision of the tumor with a small portion of the bladder wall) accompanied with BCG therapy (immunomodulation with attenuated *Mycobacterium tuberculosis*) or topical (intravesical) chemotherapy. The most important prognostic factor is the stage of cancer. With advanced stages (muscularis propria infiltration), cystectomy is routinely implemented with systemic chemotherapy.

In the present study, we report the results of an investigation on different urothelial cell lines. Importantly, each of the chosen cell lines reflects a different cancer stage, grade, luminal or basal type as well as a different genetic mutation. The T24 cell line is described as “contaminated” and has a few subtypes. As a control, we used HCV-29, which stems from non-cancerous urothelium and was immortalized by the HCV (Hepatitis C Virus) vector. T24a and T24p came from papillary, non-invasive, high-grade cancer with HRAS, TERT and p53 mutations. The subepithelial invasion, low-grade cancer with TERT, CDKN2A (cyclin dependent kinase inhibitor 2A gene, also known as protein 16 (P16)) and TP53 abberations is characteristic of the RT4 cell line. Two cell lines, HT-1376 and J82, are high grade and represent advanced stages of BC. HT-1376 has FGFR-3 (FGF3 receptor), PIK3CA (Phosphatidylinositol-4,5-Bisphosphate 3-Kinase Catalytic Subunit Alpha), TERT and p53 mutations and stems from muscle-invasive cancer, whereas J82 stems from cancer infiltrating tissues around the bladder and has PIK3CA, TERT and p53 mutations (Table 1). The literature on human BC revealed a small number of low stage (only 12 out of 127 commercially available ones) and low-grade cell lines (8/127) due to difficulties in culturing low-grade tumors in vitro; therefore, there is a clinical need for late stage and high-grade models [6]. The molecular analysis uncovered loss of or aberrant RB expression in HT-1376 and J82 and the absence of E-cadherin in J82. CDKN2A mutation interferes with RB through CDK4/6 (cyclin dependent kinase 4/6). Due to growing in the so-called “morules”, the RT4 line is a potential model of hypoxia and chemoresistance [7]. There are 127 commercially available urothelial human cell lines [6]. Most of the research was performed on high-grade lines under in vitro and in vivo (organoid models) settings, and others were used in order to study cell signaling [8]. The literature depicts BC molecular and metabolic changes investigated with a broad spectrum of methods, such as “Blotting”, “OMICS”, high performance liquid chromatography (HPLC) and spectroscopies (i.e., mass spectroscopy (MS), nuclear magnetic resonance (NMR)). MS and NMR analyses of urothelial cell lines, bladder tissues and biofluids have shown changes in, e.g., glycolysis, pyruvate metabolism, tricarboxylic acid cycle (TCA cycle), pentose phosphate pathway (PPP), as well as a level of amino acids, glutathione, triglycerides and carbohydrate metabolism alterations [9,10].

FTIR and Raman (RS) spectroscopies are methods of vibrational spectroscopy which collect a specific molecular signature of all biological systems (e.g., cells, tissues, body fluids), showing the presence of fundamental biomolecules such as proteins, nucleic acids, lipids and carbohydrates. These methods detect vibrations of functional groups due to the absorption of IR photons or Vis/NIR light scattering, respectively, which are presented as spectra. FTIR and Raman spectroscopy are complementary in information gathered from spectra. For instance, FTIR is sensitive to proteins, esterified lipids, carbohydrates and nucleic acids (in particular the secondary conformations of these molecules), while RS is more specific for the presence of hemoproteins, lipids, nucleotides and amino acid residues [11]. An enormous advantage of vibrational spectroscopy is its imaging capability, which provides a label-free and non-destructive distribution of biocomponents in cellular compartments, importantly, with a spatial resolution of up to 0.3 and 3 μm for RS and FTIR imaging, respectively. In addition, semi-quantitative assessment of the content of chemical groups is possible [11,12]. Spectral differences in bio-samples and their interpretation are usually supported by chemometric methods, particularly with the use of Principal Component Analysis (PCA) and cluster analysis (CA). PCA reduces the dimensionality of a data set to find interrelated variables and to retain the variation present in the spectral data set collected for various experimental groups. Each object is characterized by two parameters—scores (spectral properties of samples) and loadings (the relationship between variables). PCA determines the principal components, which are a linear combination of studied variables. CA is in turn a method for the examination of a hyperspectral data set acquired in IR and Raman imaging. Here, an object is understood as a single pixel spectrum in the image. As a result, clusters (classes) are determined, in which objects in one cluster possess similar spectral features and are different as much as possible from elements in other clusters. Pixels from each class are coded into a color, providing a false-color map. A spectral profile of the cluster is represented by an average spectrum. Grouping into classes is based on a defined mathematical algorithm. Unsupervised Hierarchical Cluster Analysis (UHCA), commonly used in IR imaging, relies on a distance between objects, whereas k-means cluster analysis (KMCA) employed for the analysis of Raman images is based on intraclass variance.

Until recently, several reports showed the applicability of FTIR and RS for the examination of bladder cancer cells. For example, PCA analysis of Raman spectra distinguished MGHU (an urothelial BC line) from prostate cancer cell lines (BPH, PC3 and LNCaP), mostly on the basis of the data coming from proteins (lower signal in BC), and indicated that the BC cell lines are characterized by a lower content of proteins and elevated levels of nucleic acids and lipids in comparison to prostate cancer [13]. The T24 and RT112 urothelial cell lines were discriminated by their Raman spectra supported by neural network and PCA algorithms [14,15].

Moreover, Raman studies by Chun-Ping and co-workers revealed keratin-like structures accompanied with a high level of proteins and lipids in a normal urothelium (E7 cell line) in contrast to cancer cells (TSH-8301, J82 and TCC-SUP), which showed an increased content of tryptophan residues [16]. Canetta et al. found a higher content of DNA in the BC MGH-U1 cell line in comparison to the normal urothelial SV-HUC cell line [17].

FTIR and Raman-based classification of urine and bladder tissues also showed strong evidence of a potential application of these methods for the diagnosis of patients and as useful tools for research on the mechanism of carcinogenesis. For instance, attenuated total reflection (ATR) and transmission FTIR spectra of a urinary bladder wash from 163 patients, by revealing lipid and carbohydrate bands specific for normal or cancer cells, enabled segregation between healthy and cancer groups with a specificity and sensitivity of 82–100 and 59–81%, respectively [18]. Yosef et al. reported that the glycogen to oleic acid ratio, determined in Raman spectra of cells from patient urine samples, is higher for the control than the neoplasm group [19]. Although the glycogen level was found to be higher in the normal urothelium than the cancerous one, a very small number of cancer cell types contained glycogen and only one FTIR feature cannot be used to detect all cancer types [20]. Shapiro and co-workers proposed that the Raman band at 1584 cm^−1^ assigned to hemoproteins is a hallmark of urothelial carcinoma and can be correlated with cancer grade, with a specificity and sensitivity of ca. 90% [21]. RS imaging of cells in the bladder tissue discriminated normal tissue, cystitis, cancer cells in situ (Tis), urothelial bladder cancer of various grades (G1, G2, G3) and stages (T1, T2, T3) as well as adenocarcinoma [22]. The semi-quantitative analysis of the bladder tissue showed an increase in cholesterol and choline levels, with a simultaneous decrease in collagen content during carcinogenesis, while DNA, oleic acid, and triglycerides were increased at early stages of cancer development and then their levels slowly fell down [23].

The penetration of genes affects metabolomic and subsequently morphological changes, both in vitro and in vivo, and it can be supported by advanced molecular spectroscopic imaging performed using vibrational spectroscopy. Therefore, the aim of this work was to determine biochemical alterations in BC cell lines derived from different cancer stages and grades to evaluate their diversity and to assess whether biological aggressiveness can be interrelated with chemism of the BC cells revealed by molecular spectroscopy (Table 1). Reports published so far [13,14,15,16] showed the powerful ability of FTIR and Raman spectroscopic imaging for the identification of the BC cells in some groups; however, there is still a lack of high-definition microscopic visualization of the cellular environment which would give an insight into the metabolic abnormalities of bladder cancer.

For this purpose, we employed a technique that is simple in operation and rapid, i.e., ATR FTIR, to establish biomarkers for urothelial carcinoma and, additionally, cells were imaged with high-spatial resolution FTIR and Raman microscopy to depict molecular differences between the nuclei and cytoplasm. We intend to provide a spectroscopic profile of human bladder cancer cell lines derived from different stages, which would be further utilized for the application of these models in pharmacological studies and for the development of a proper classification, necessary for an accurate identification of cancer cells in patient urine sediments and tissues.

## 2. Results

### 2.1. Morphology of Cells

**Figure 1:**
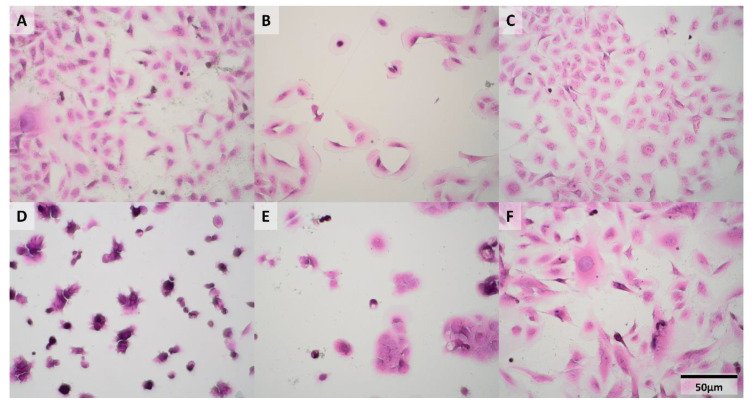
Photographs (100×) of the investigated cell lines stained with hematoxylin and eosin (HE): (**A**)—HCV-29, (**B**)—T24a, (**C**)—T24p, (**D**)—RT4, (**E**)—HT-1376, (**F**)—J82.

Hematoxylin-eosin (HE) stained cells are presented in Figure 1 and reveal a plethora of morphological features. The higher the stage of derivation of the cell line, the higher the polymorphism (dispersion of cell features) that was noted. Some of these cell lines possess two nuclei, typical for urothelial cells. The RT4 cells had a smaller diameter than others and formed “morules”. We noticed that some of the HT-1376 cells were grown in clusters, whereas other ones displayed a similar morphology to RT4. Moreover, HT-1376 and RT4 exhibited irregular nucleoli and small vacuoles. J82 and RT4 had different shapes and diameters of cells and nuclei, and it seemed that a part of the cell was attached well (such as pseudopodia) and another was not. The cytoplasm of invasive RT4, HT-1376 and J82 cells was much more eosinophilic and the nuclei were bluer (so-called hyperchromasia; small pink nucleoli are poorly found) than in the non-invasive ones.

**Figure 2:**
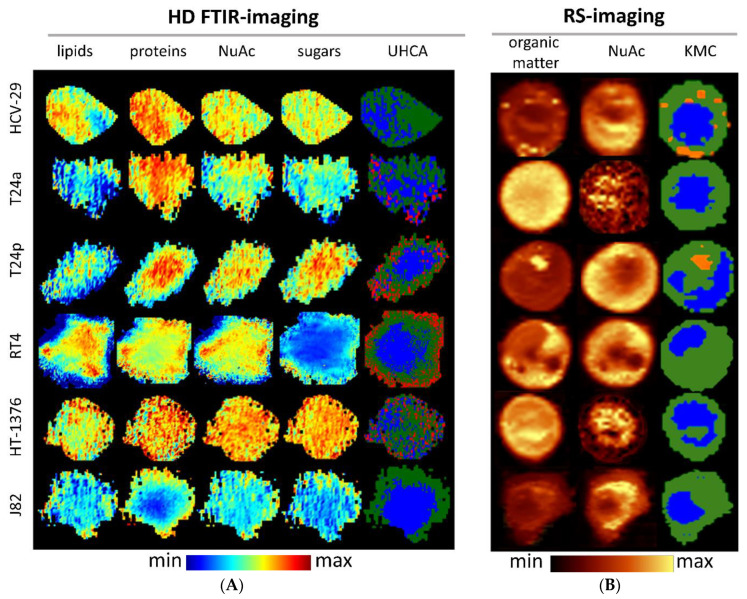
Examples of HD FTIR (**A**) and RS images (**B**) constructed for integral intensities of bands specific for main cellular macromolecules and their clustering into false-color maps (Unsupervised Hierarchical Cluster Analysis (UHCA) and k-means cluster (KMC) for FTIR and Raman images, respectively). The colors in UHCA and KMC cluster maps correspond to nucleus (blue), cytoplasm (green), graininess in cytoplasm (red; present in all 20 cells of the four cell lines except HCV-29 and J82), and lipid droplets (orange; present in 18 out of 20 HCV-29 cells and 17 out of 20 T24p cells). Abbreviations: HD—high definition, NuAc—nucleic acids. Color bars (min–max) denote changes in intensities of bands chosen to construct chemical images.

High-definition (HD) FTIR and Raman spectroscopic imaging was performed with a spatial resolution of ca. 1 µm. Spatial resolution for HD FTIR imaging is wavelength-dependent (a light source emits IR radiation and detects it in the region of 900–4000 cm^−1^) and limited by the pixel size of the focal plane array FPA detector illuminating some field of view, that was 1.1 μm × 1.1 μm. So spatial resolution is c.a. 3.0 μm for 2500 cm^−1^ and 10 μm for 1000 cm^−1^. In the case of Raman imaging, the sampling density was 0.3 µm giving 0.9 µm of spatial resolution according to the Nyquist sampling theorem. Then, the distribution of biomolecules was presented as chemical images and false-color maps clustering pixels with similar chemical signatures. The nuclei were separated from the cytoplasm by an increased intensity of DNA bands at 1240 cm^−1^ (IR), 785 (RS) and 835 (RS) cm^−1^. In both the HCV-29 control and advanced-stage cells (muscle invasive BC lines: HT-1376 and J82), the distribution of biocomponents was irregular, although it should be noted that this was not the case for all biocomponents, e.g., proteins in J82 are spread out to the borders of the cell. We assumed that enzymes which are responsible for tissue matrix degradation are overproduced and their removal is disturbed [24]. The irregular distribution of cellular compartments was reported for cancer cells and their stability may depend on the cell phenotype and their chemical content [25,26]. Another possibility of cellular structure change is the dysfunction of cellular tethers [27]. However, cells from lower stages of cancer (T24a, T24p and RT4) exhibited regular distribution of biocomponents concentrated in a close proximity to the nucleus, except sugars in the RT4 cell line that are localized on the periphery of cells. It is worthy to stress here that Raman imaging detected numerous lipid droplets in most HCV-29 and T24p cells, see the KMC maps in Figure 2 in contrast to HE staining which is not sensitive to lipid fractions. Their Raman features were specific for lipids since they are good Raman scatterers and appeared at 2852, 1660, 1447, 1307, and 1268 cm^−1^. However, the clustering of FTIR images differentiated some small structures which we called “graininess” and was not clearly detectable neither by hematoxylin and eosin nor reported in previous studies. These cellular structures were hardly visible in HE stains because they were small and similar to artifacts, whereas staining is pH-dependent and graininess might be washed out during staining. Enlarged HE images of cells were depicted in Figure S1 in Supplementary Materials (SM) and showed potential localization of these structures in the cytoplasm. Figure S2 in SM displayed all FTIR spectra of cellular structures extracted from UHCA analysis of FTIR images to show their high S/N ratio. We described their chemical composition in the next section.

### 2.2. FTIR-Based Biocomposition of BC Cells

**Figure 3:**
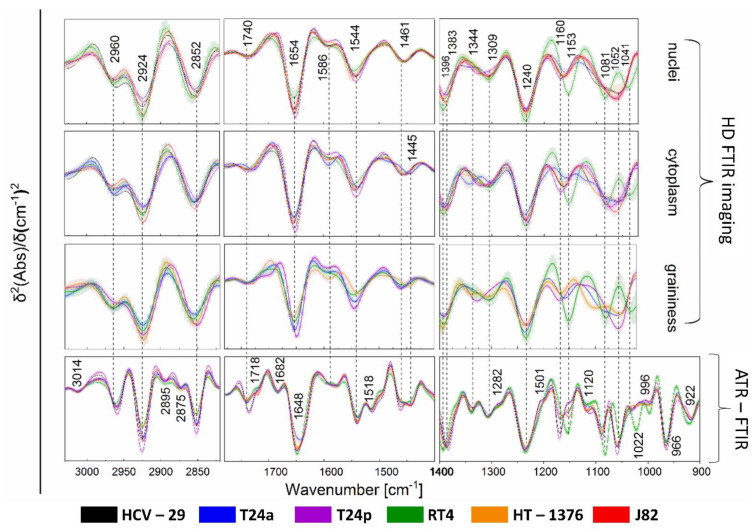
Averaged second derivatives of FTIR transmission spectra of the nuclei and cytoplasm for each cell line and graininess observed for T24a, T24p, RT4 and HT1376 cells compared to ATR−FTIR spectra of whole cells. Gray shading denotes standard deviation (±SD); N = 20 spectra per cell line. Averaged normal FTIR spectra are shown in Figure S3 in SM.

To highlight the differences in the biochemical composition of the studied cells, one can examine the positions and intensities of the bands, which are proportional to the content of the biomolecules. The FTIR spectra, derived from cellular organelles differentiated by cluster analysis (UHCA), are displayed in Figure 3 and Figure S3, while the band assignment to biomolecules is summarized in Table S1 [12,28,29,30,31]. Below, we outlined the most evident spectral differences observed in the FTIR spectra of cellular compartments and calculated integral intensities of selected bands, as shown in Figure S4 in SM.

An analysis of the high-wavenumber region of FTIR spectra showed an increased intensity of the 2924 cm^−1^ band assigned to the CH_2_ moieties in the alkyl chain of fatty acids in RT4 and J82 nuclei (Figure S4aA). This signal decreased in the order from HCV-29, HT-1376, T24p, to T24a cell lines. On the other hand, the CH_2_/CH_3_ ratio (2924 cm^−1^/2960 cm^−1^) indicated that the nuclei of control bladder cells (HCV-29) contained longer acyl chains of fatty acids than low-grade BC cells, but these chains were significantly elongated in invasive and high-grade BC cells (Figure S4aB) [12,26]. In addition, a shift of the COO^-^ band from 1396 cm^−1^ in the spectra of RT4, HT-1376 and T24a cell lines to 1383 cm^−1^ for other cells indicated that the composition of free fatty acids also varied and was independent of the stage and grade of BC (Figure S4aC). An elevated level of these biomolecules was observed for nuclei of the low-grade RT4 cells, contrary to proteins whose amide I and II bands (1654 and 1544 cm^−1^, respectively) exhibited low intensity (middle panel in Figure 3). This trend is also reflected in the total amount of proteins, which is the lowest in RT4 in contrast to T24p (Figure S4aD). The highest content of DNA highlighted by the intensity of the 1240 cm^−1^ band was found for low-grade RT4 cell lines (Figure S4aE). The nucleus of this cell line also showed a specific spectral profile in the region of sugars. Bands at 1153, 1081 and 1041 cm^−1^ indicated the presence of glycogen and other carbohydrates (right panel in Figure 3). This short FTIR-based characterization of BC nuclei indicated that the low-grade cell line was distinct from high-grade cancer cells.

The lipid profiles in the high-wavenumber IR region of the cytoplasm were found to be specific for each cell line (left panel in Figure 3). Both signatures of the CH_2_ (Figure S4bA) and CH_3_ (Figure S4bB) moieties differentiated control and BC cells that could result from the presence of lipid droplets indicated by Raman imaging. Moreover, the FTIR spectra indicated the presence of triacylglycerols by a band at 1740 cm^−1^ (middle panel in Figure 3). Their content was elevated in the T24p cell line (Figure S4bC), whereas its genomic subtype T24a additionally contained cholesterol esters (1735 cm^−1^) [12]. The protein level in the cytoplasm was similar to the spectral pattern found for nuclei (Figure S4aD,bD). In the spectrum of T24a cells, a shift of the amid III band from 1309 to 1300 cm^−1^ was observed, which resulted from the presence of polarized groups attached to proteins or the cross-linking of proteins with highly polarized biocomponents. The spectral region below 1400 cm^−1^, which showed vibrational modes of carbohydrate, phospholipid, and nucleic acid bands, is specific for each cell line (right panel in Figure 3). In particular, carbohydrates and polysaccharides differentiated the cell lines. The intensity and position of bands at ca. 1160 cm^−1^ assigned to lipid esters confirmed our previous observations and present the highest content for RT4 cells (Figure S4bE). Glycogen was present only in the T1 cell line, while an elevated level of RNA (1120 cm^−1^) was found in the cytoplasm of T2 BC cells. A signal with a maximum at ca. 1055 cm^−1^ varied regardless of the stage and grade of BC and its alternation may be indicative of carbohydrate, protein and lipid biocomponents from cerebrosides which stabilize the urothelial cell membrane [29,32].

Cytoplasmic graininess was identified by HD FTIR imaging in BC cells in stages Ta, T1, and T2 (Figure 2). Each of them showed an individual set of spectral features in the entire region of the FTIR spectrum (Figure 3). Briefly, the lipid level increased along with the aggressiveness of the cancer cells (Figure S4cA) and contrary to the protein content, where the less aggressive cells displayed a tendency for their accumulation (Figure S4cB). In addition, the position of the amide I band shifted from 1654 cm^−1^ for the T2 stage to 1647 cm^−1^ for Ta, suggesting α-helical conformations of proteins dominating in T24a, RT4 and HT-1376 cells, whereas unordered structures were characteristic for T24p. A complex variation in bands below 1400 cm^−1^ also indicated a significantly different composition of sugars and nucleic acids. For instance, the DNA level was higher for low-grade BC than for the high-grade ones (Figure S4cC), whereas the RNA band at 1120 cm^−1^ appeared in the spectra of the high-grade T24a and HT-1376 cells. Sugar moieties linked with lipids and proteins were present in graininess of the high-grade BC, contrary to the low-grade RT4 cell lines that again contained glycogen.

The attenuated total reflection FTIR technique (ATR FTIR spectroscopy) is a rapid alternative to FTIR imaging by which IR spectra of cell sediments can be recorded. That way, we collected the spectral characteristics of whole cells with a higher spectral resolution than using HD FTIR microscopy and in a wider spectral region up to 900 cm^−1^ (Figure 3). In principle, the ATR FTIR spectra confirmed the observation from FTIR imaging that each cell line exhibits a unique IR signature. Again, the most distinctive spectral profile was observed for the low-grade RT4 cells, in particular in the region of glycogen bands at 1153, 1081 and 1022 cm^−1^. Lipid bands revealed prominent spectral markers found in the ATR FTIR spectra, the presence of the 1740 cm^−1^ band (triacylglycerols) in the spectra of HCV-29 and T24p and a band at 1735 cm^−1^ attributed to esters of cholesterol and cerebrosides. The latter constitute lipid-carbohydrate components present in cellular membranes and are responsible for the stabilization of uroplakin rafts, flexibility and permeability. Differences in the ratio of lipids and proteins were indicated by bands at 1461 and 1455 cm^−1^, respectively. Here, we noticed that this ratio is elevated for the control bladder and T24p cells. Moreover, high-stage BC lines (J82 and HT-1376) showed the highest intensity of the 1718 cm^−1^ band assigned to the C=O vibration base pairs in DNA, probably resulting from hyperploidy or altered activity of DNA due to epigenetic gene activation.

### 2.3. Raman-Based Biocomposition of BC Cells

**Figure 4:**
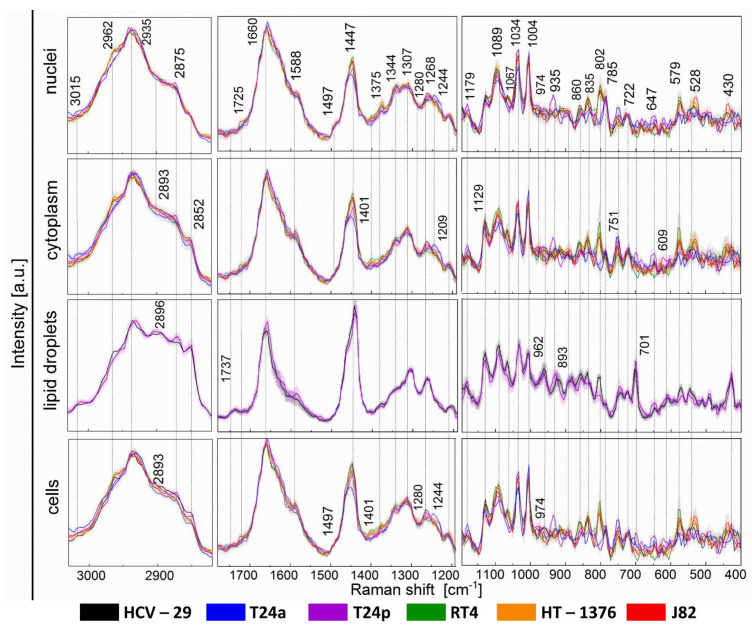
Averaged Raman spectra of whole cells, nuclei, cytoplasm, and lipid droplets (only for HCV-29 and T24p) calculated in KMC analysis for each cell line. Gray shading denotes standard deviation (±SD); N = 20 spectra per cell line.

K-means cluster analysis of Raman images provided mean Raman spectra extracted for the nuclei, cytoplasm and lipid droplets (Figure 2 and Figure 4, Table S2 [13,16,33,34,35,36]). Changes in band intensities were summarized in Figure S5 in SM.

Nuclei in Raman images were mainly recognized by the presence of bands at 785 and 835 cm^−1^ (vibrations of the phosphate group of nucleic acids and B-DNA) and their increased intensities correlated with a marker band at 1375 cm^−1^ attributed to deformations of the CH_3_ groups in adenosine (Table S2). The intensities of the three RS nuclei markers increased in the following order: T24p/T24a, J82, HCV-29, RT4, and HT-1375 (Figure S5aA; showed for the 835 cm^−1^ band). The shape of the low-wavenumber bands suggested that DNA in the Ta cell lines was methylated, since the 785 cm^−1^ band is single and narrow, and accompanied by very small intensity bands at 820 and 835 cm^−1^ [37]. Other bands in the Raman spectra of the nuclei indicated that the variation in the chemical environment around the DNA chromatin resulted from different contents of phospho/lipids (802, 1067, 1089 cm^−1^, Figure S5aB–aD), cholesterol and its esters (430, 1725 cm^−1^, Figure S5aE), carbohydrates (578 cm^−1^, Figure S5aF) as well as various forms of protein systems (Figure 4). As for the last ones, we found bands for the *gauche-gauche-trans* conformation of the disulfide bond (528 cm^−1^, Figure S5aG), amino acid residues (Tyr: 1179 cm^−1^, Phe: 1004 cm^−1,^ Figure S5aH), protein crosslinking (1034 cm^−1^ Figure S5aI), and secondary structures of proteins (amide III: 1244 and 1268 cm^−1^ for extended and helical conformations, respectively) (Figure S5aJ; showed for the 1244 cm^−1^ band). The intensities of these bands differed between the cell lines, e.g., phospholipid (Figure S5aB–aD), cholesterol (Figure S5aE) and S-S bands (Figure S5aG) are of the highest intensity for the most invasive T2 and T3 cells. Phe and amide III bands (Figure S5aH,aJ) at 1004 and 1244 cm^−1^, respectively, separated non-invasive BC cells from others. Interestingly, the two cell types in the Ta group showed a completely different level of protein crosslinking (1034 cm^−1^, a low and high intensity for T24a and T24p, respectively (Figure S5aI)), whereas the disulfide bridges in the nuclear proteins were absent from both cell lines. This could be associated with the presence of a keratin-like structure in uroplakin, which was found only for T24p cells (935 cm^−1^).

The Raman features of the cytoplasm indicated an increase in the lipid level in comparison to the nucleus (2852 cm^−1^) and revealed the presence of cytochromes, (1588 and 751 cm^−1^), cf. Figure 4. A level of reduced cytochrome c and b is higher in cells containing cholesterol (Figure S5bA), whereas the other band of cytochrome c exhibits high intensity in the T24a cell line. The lowest accumulation of predominantly c than b isoform of cytochrome was observed for the low-grade RT4 cell line that, in contrast, exhibited the highest level of carbohydrates (Figure S5bB; showed for the 579 cm^−1^ band) and phospholipids (Figure S5bC; showed for the 802 cm^−1^ band). These bands were absent in the spectra of the cytoplasm in the Ta cells (Figure 4).

Raman imaging revealed the presence of lipid droplets (LDs) in healthy bladder cells (HCV-29) and in T24p cells from the Ta stage (Figure 2 and Figure 4). In general, the Raman spectra of LDs indicated that mainly fatty acids and triacylglycerols contributed to their composition (Figure 4). A careful examination of these spectra implied significant differences between these cell lines. Fatty acids in HCV-29 cells were less unsaturated than T24p (3015, 1660 cm^−1^) and were mostly arranged in the *gauche* conformation (1067 cm^−1^). Interestingly, phospholipids were present only in the LDs of control cells (802, 1089 cm^−1^) and there, one can find an increased content of carbohydrates (579 cm^−1^), Tyr (835 cm^−1^), and S-S bridges in proteins (528 cm^−1^). In contrast, unsaturated fatty acids in the LDs of T24p cells were accompanied by cholesterol and its esters (701 cm^−1^) as well as cytochromes (1588, 751 cm^−1^).

We also compared averaged Raman spectra for whole cells as we had done for FTIR measurements. However, they were dominated by the cytoplasm profile and did not show additional chemical information (data not shown).

We summed up the cellular biocomponents observed spectroscopically in Table 2.

### 2.4. In Search of a Spectroscopic Model of Bladder Cancer Cells

Cluster and Principal Component analyses (CA and PCA, respectively) are well-established methods suited for distinguishing subtle spectra variations in large data sets. The obtained segregation of groups indicates a potential of FTIR and Raman spectroscopy for further classification of cells in a complex matrix of cytological samples or tissues.

**Figure 5:**
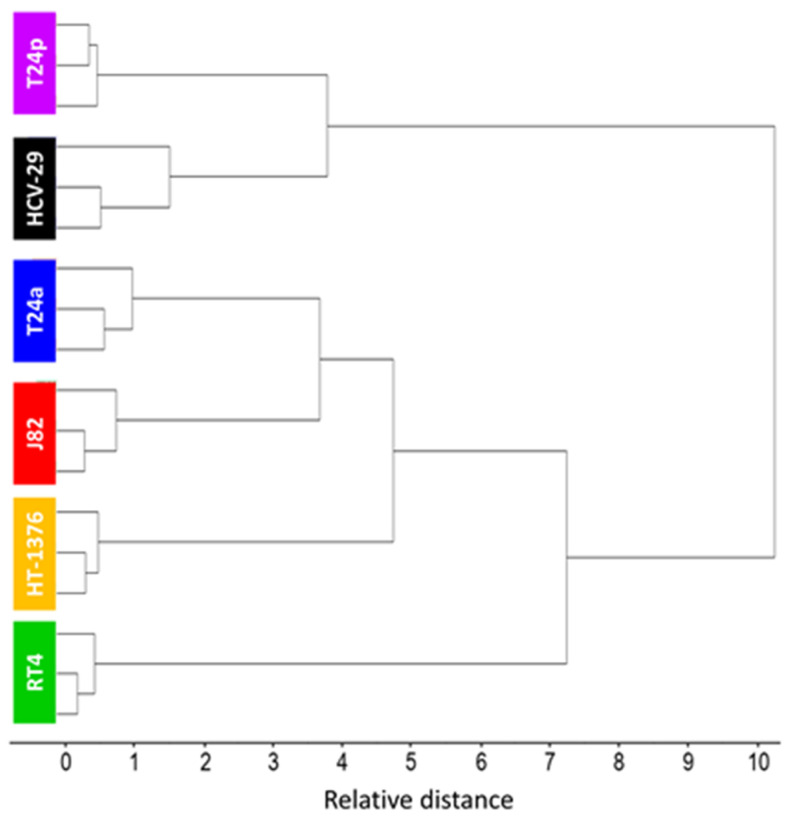
Hierarchical Cluster analysis of ATR−FTIR spectra using Ward’s squared Euclidean distance. Analysis performed on second derivative spectra in the bio-regions of 1020–1780 and 2820–3030 cm^−1^, N = 3 spectra per cell line.

The ATR−FTIR spectra of cell sediments collected in the simplest possible way for three independent replicates of cultures of urothelial cell lines were evaluated with the use of hierarchical cluster analysis in the bio-region including the fingerprint (1020–1780 cm^−1^) and high-wavenumber regions (2820–3030 cm^−1^), see Figure 5. This means that the ATR−FTIR spectrum represented the total biochemical composition of the cells and indicated molecular differentiation of the cell lines at a level of the whole cell. The CA dendrogram showed a strong differentiation of two groups. The relative distance of this distinction is much higher than the differences between replicates, indicating that the ATR−FTIR signature of each cell line in three replicates was reproducible, as is also visible in average spectra with their SD displayed in Figure S3 in SM. In the first group, the HCV-29 control was clustered with the non-invasive papillary type of BC—T24p, but both cell lines were further divided into sub-groups. The second CA group included other cell lines further divided into three branches. The most distinctive cell line in terms of the ATR−FTIR profile was the low grade, non-muscle invasive bladder cancer (RT4) that infiltrated subepithelial tissue. This was congruent with the FTIR-based biochemical composition of the cells discussed above. The high-grade non-invasive T24a was grouped close to the high-grade invasive J82 cells, although they exhibited their own spectral profile. Surprisingly, T24a and T24p cell lines that stem from one culture were not clustered together, which could indicate an effect of epigenetic changes between them that affect their overall spectral characteristics. This result clearly implicated that BCa cells exhibited specific spectroscopic features.

**Figure 6:**
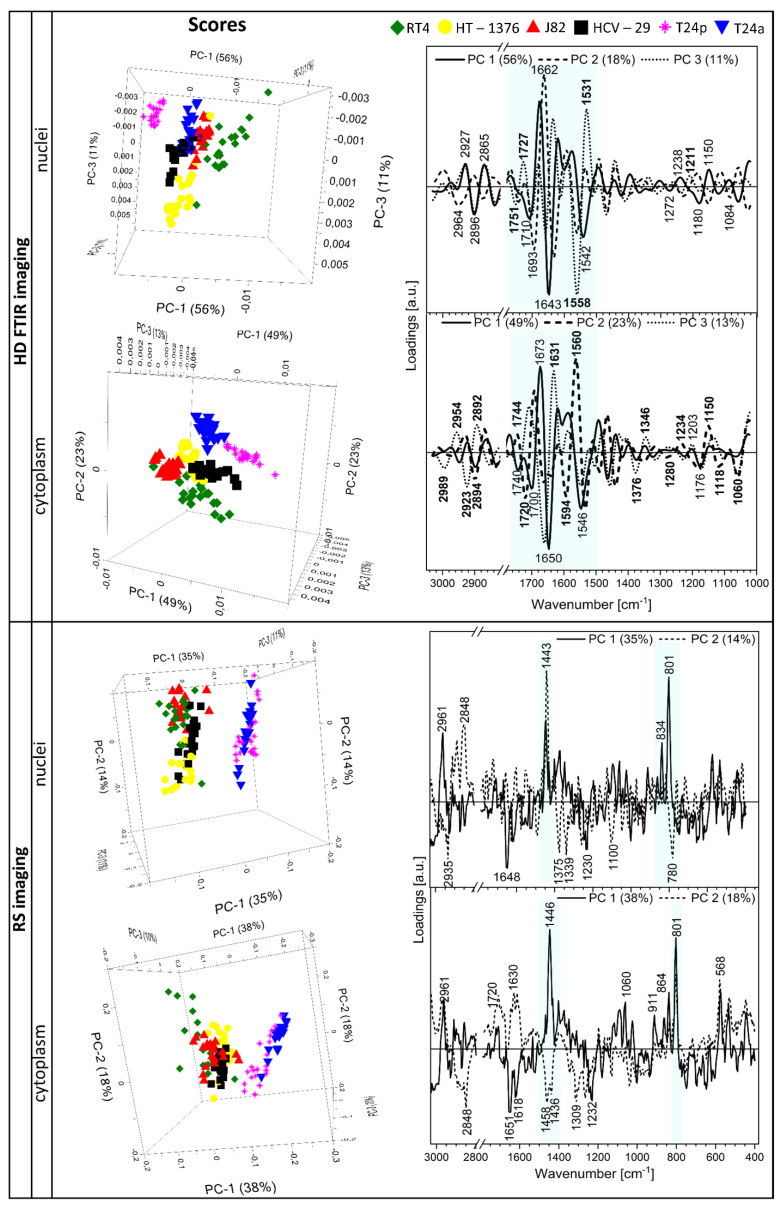
Three-dimensional PCA score plots for the nuclei and cytoplasm of the cell lines derived from UHCA and KMC analyses of HD FTIR and RS images (**left**) and their respective loading plots for PC 1–3 (**right**). Analyses were performed on second derivative FTIR spectra and Raman spectra in the bio-region. The aqua color in the loading plots highlighted the strongest discriminators. The band assignment was summarized in Tables S1 and S2.

Since high-resolution FTIR and Raman imaging provided the information about the nuclei and cytoplasm of cells, PCA was performed on spectra of both cellular compartments. Three-dimensional score plots along principal components 1–3 and their loadings are displayed in Figure 6. All score plots showed grouping of the investigated cell lines in a distinct manner, indicating that FTIR and Raman spectra of the nuclei and cytoplasm can deliver classifications of various sensitivity for particular groups of bladder cancer.

The score plot for the FTIR-transmission spectra of nuclei showed that the spectra projected on PC-1 with a high variation of 56% segregated RT4 cells (T1 stage) on negative PC-1 and T24p cell line (Ta stage) on positive PC-1 (Figure 6). This suggested that chemism of the RT4 and T24p nuclei was specific for these cell lines. Positive loadings on PC-1 at 2927, 2865, 1673, 1238, and 1150 cm^−1^ were mainly attributed to lipids and nucleic acids, whilst negative loadings at 2896, 1710, 1643, 1542, 1180, and 1084 cm^−1^ were largely assigned to proteins, fatty acids, cholesterol esters, and phospholipids, respectively, see Table S1. Given that PCA was calculated on second derivative FTIR spectra and RT4 cells are negatively loaded on PC-1, lipids and nucleic acids expressed by positive values of loading vector (*ve+*) on PC-1 indicated that they are more abundant in the nuclei of these low-grade bladder cancer cells than in other cell lines. For a better visualization of the spectral projection on PC axes, 2-dimensional score plots are displayed in Figure S6 in SM. The corresponding PC-1 vs. PC-2 plot for the nuclei showed that the J82 nuclei (T3) were also located with RT4; however, this group was close to the center of PCA axes, so its spectral features do not contribute strongly to negative PC-1 scores. PC-2 scores (variation: 18%) indicated differentiation between RT4/T24p (positive PC) and J82 cell lines (negative PC) whilst PC-3 (variation: 12%) differentiated the HT-1376 nuclei (T2 stage) from other groups. The key features from positive PC-2 originated from turns and sheets of proteins (2964, 1693, 1630, 1558 cm^−1^), whereas HT-1376 cells differed by bands at 1650 and 1542 cm^−1^ attributed to α-helical conformations of proteins (Figure 6).

The PCA analysis of FTIR spectra extracted from cytoplasm showed different grouping of the cell lines compared to nuclei (Figure 6 and Figure S6). Principal component 1 showed grouping of control and T24p cells on positive PC-1, whilst negative PC-2 scores segregated RT4 cells from other cell lines. In the case of PC-1, α-helices in proteins (1650, 1546 cm^−1^), sugar moieties (1060 cm^−1^), and esterified fatty acids and cholesterol (1740, 1176 cm^−1^) gave largely negative loadings specific for HCV-29 and T24p cytoplasm, whereas turns of secondary structures of proteins (1690, 1560 cm^−1^) and glycogen (1150 cm^−1^) are more abundant in RT4 cells. On the other hand, PC-3 grouped all cell lines separately. Here, key features determined from positions of loadings were mainly attributed to β-sheets (*ve+*: 1631 cm^−1^) and turns (*ve*−: 1660 cm^−1^). Contrary to FTIR-based PCA analysis, the PC-1 component with a variation of ca. 35% of the score plots for Raman spectra of the nuclei and cytoplasm showed segregation of T24a and T24p cell lines—papillary non-invasive BC versus other cell lines, cf. Figure 6. In addition, PC-2 for nuclei separated RT4 and J82 cell lines from HT-1376. DNA and phospho/lipid moieties were found to be more dominant in nuclei of the non-invasive BC cells (801, 834, 1443 cm^−1^) than in other cell lines, whereas positive PC-2 loadings for RT4 and J82 cells were given for more abundant lipids (2848, 1443 cm^−1^) than in HT-1376. The last one was distinguished mainly due to the presence of Raman features of nucleic acids bases (785, 1339, 1375 cm^−1^). On the other hand, the Raman spectra of the cytoplasm in the T24a and T24p cell lines were grouped together due to the presence of discriminators at 1651, 1618, and 1232 cm^−1^ assigned to proteins (Figure 6). Carbohydrates and lipids mainly contributed to the differentiation of the cytoplasm from other cell lines (Figure 6). 

In addition, we performed Partial Least Squares Discriminant Analysis (PLS DA) using HD-FTIR and RS spectra of the nuclei and cytoplasm of carcinoma and normal urothelial cells (Figures S7–S10, Table S3 in SM). Regression models from the calibration and validation and prediction sets had correlation coefficients of ca. 0.9, which showed that datasets were very well modelled. The classification for two independent validation dataset groups was applied to calculate the prediction accuracy of the PLS-DA model with almost 100% of spectra correctly assigned to the normal urothelial cells and those derived from bladder carcinoma. Observed loadings of factors were congruent to our findings in PCA.

## 3. Discussion

Quantitative and qualitative changes in nucleic acids of BC cells might be caused by epigenetic alterations, i.e., de/activation of genes by the addition of hydrophobic or hydrophilic chemical groups to nucleic acids and their proteins. After some time, immortalizing mutations appear in cells and turn them into “selfish busters”, which can be compared to cancer in situ or a papillary BC type, as in T24a and T24p cell lines that were clearly distinguished in PCA of their Raman spectra (Figure 6). Phosphatidylinositol 3-kinase (PI3K), a mutation present in the advanced stage HT-1376 and J82 cell lines, activates protein kinase B (PKB also known as AKT), which in turn results in an increase in glucose uptake and glycolysis [38]. A metabolic switch to glycolysis (the Warburg effect) results in a decrease in glycogen content, increased lipid content and its metabolism as well as an increase in proteinase production [39]. In our results, this switch was observed through the presence of the FTIR glycogen bands in the T1 cells (RT4) and their absence in cells from the advanced stages of BC (Figure 3). Raman spectra of the cytoplasm clearly showed elevated levels of cytochromes in J82 and RT4 cells, which could be associated with their activity in mitochondria towards the production of free reactive oxygen species (ROS) and acceleration of the Warburg effect [40] (Figure 4). This signal can be used to give an insight into oxygen stress and facilitate investigations of therapeutic metabolism. Moreover, viral infections such as hepatitis C, used to obtain the control HCV-29 urothelial line, can induce changes in lipid metabolism via the mitochondrial system, and furthermore, alterations in the catabolism of carcinogenic factors also occur [40]. Our RS study revealed the presence of lipid droplets in the T24p and HCV-29 lines, and these droplets are composed of long fatty acids, cholesterol, triglycerides and phospholipids (Figure 2 and Figure 4). Altered lipid metabolism affects the mevalonate pathway and induces cholesterol production [41]. An elevated level of cholesterol esters appears with mutations in the kinase genes of phosphatase and the tensin homolog (PTEN)/PI3K3/mTOR) pathway, which are correlated with a higher stage and Gleason score in prostate cancer cells. The depletion of the cholesterol ester pool leads to a reduction in cancer proliferation and invasion capability in mouse xenograft models with minor toxicity [42]. The presence of S-S bridges in proteins observed for aggressive J82, HT-1376 and RT4 cancer cells may be due to the elevated oxygen stress and NAD^+^ level, TCA cycle, PPP pathway, and inhibition of ferroptosis mechanisms [9,43] (Figure 4).

## 4. Materials and Methods

### 4.1. Cell Culture and Sample Preparation for Spectroscopic Measurements

Human bladder urothelial cell lines were obtained from ATCC and certified Mycoplasma free: HCV-29, T24a and T24p (HTB-4™), RT4 (HTB-2™), HT-1376 (CRL-1472™) and J82 (HTB-1™). All the cell lines were epithelial and adherent. Cells were cultured according to the ATCC protocol and the manufacturer recommendations: HCV-29—RPMI 1640 medium (Sigma Aldrich, Warsaw, Poland); HT1376, J82—Eagle’s minimum essential medium (MEM Gibco, Thermo Fisher); T24a, T24p, RT4—McCoy’s 5a modified medium (Sigma Aldrich). In addition, the media were supplemented with 10% fetal bovine serum (FBS, Gibco, Thermo Fisher, Grand Island, NY, USA), 50 U/mL penicillin, and 50 µg/mL streptomycin (Invitrogen Thermo Fisher, Grand Island, NY, USA). There was no correlation between the content of media and spectral features assigned to biocomponents. Semi-confluent cell cultures were initially seeded with 1 × 10^5^ ÷ 5 × 10^5^ cells/75 cm^2^ simultaneously on CaF_2_ windows and into Petri dishes and maintained at 37 °C in a humidified atmosphere of 5% CO_2_ for 48 h. After 24 h, the culture medium was replaced with a fresh portion. For Raman and FTIR imaging, the cells cultured on CaF_2_ windows were fixed with 2% glutaraldehyde in PBS, washed with PBS and kept in PBS at 4 °C until spectroscopic measurements were performed. This fixation method was proven to have a minimal impact on molecular markers revealed in FTIR and Raman spectra [33]. ATRFTIR spectra were recorded for cell suspension; therefore, the standard protocol (TrypLE^TM^ Express, Gibco, Grand Island, NY, USA) was used to dissociate cells from the Petri dish. After spectroscopic measurements, the cells on CaF_2_ slides were stained with hematoxylin and eosin (HE) for the gold-standard histopathological examination. Examination and photographic documentation were performed using an Olympus BX53 white-light microscope equipped with an Olympus DP27 digital camera (Department of Pathology, University Hospital, Krakow, Poland).

### 4.2. FTIR Spectroscopy, Spectral Pre-Processing and Analysis

Pre-processing and chemometric analysis of the collected FTIR images were performed using CytoSpec (ver. 2.00.01) [44], MatLab (R2015a, MathWork, Natick, MA, USA), Unscrambler X software (v. 10.3, Camo, Montclair, NJ, USA), and Origin 9.1 (ver. 2018b, OriginLab, OriginLab Corporation, Northampton, MA, USA) software.

ATR FTIR spectra were collected using an ALPHA Bruker spectrometer equipped with a 1-reflection ATR diamond crystal. Three spectra of each urothelial line were collected in the range of 400–4000 cm^−1^ with a spectral resolution of 4 cm^−1^. Since the region below 900 cm^−1^ did not show spectral differences, we did not analyze it. A total of 128 scans were co-added. Before further analysis, extended ATR correction, implemented in a Bruker Opus 7.0 software, was employed. Spectra were normalized according to a vector algorithm in the region of 1780–900 cm^−1^. The second derivative IR spectra were calculated using a 13-point smoothing Savitzky–Golay algorithm. The above-mentioned processing of spectral data was performed using the OPUS software. The graphs were generated using Origin Lab 2018 Pro software. The pre-processed spectra were then analyzed using Cluster Analysis (CA) provided by Unscrambler X software for the combination of 3030–2820 cm^−1^ and 1780–1020 cm^−1^ spectral regions.

High definition FTIR-transmission images were collected from 20 cells of each cell line in the range of 900–3700 cm^1^ using an FTIR Agilent 670 spectrometer equipped with a 128 × 128 FPA camera with a pixel size of 1.1 μm × 1.1 μm, and objective 15× with NA = 0.62. To obtain these images, 1024 scans were co-added with a spectral resolution of 8 cm^−1^. All spectra in IR images after vapor removal were smoothed with a Savitzky–Golay algorithm (9 points) and vector normalized in the region of 1780–1000 cm^−1^. Quality test and PCA-based noise removal were employed with 10 PCs. Quality test was performed in the region of 1620–1680 cm^−1^. This introduced a threshold level as well as eliminated signals with absorbance lower than 0.2 and greater than 1.2. Spectral noise was reduced by performing principal component analysis (PCA) of the image data and re-assembling spectra based on low-order principal components selection (good quality spectra), whereas higher-order PCs (mainly noise) were excluded from further analysis. Unsupervised hierarchical cluster analysis (UHCA) was executed in the entire spectral region using the second derivative FTIR spectra. Spectral distances were computed as D-values and individual clusters were extracted according to Ward’s algorithm (MatLab, Cytospec). Resonant Mie scattering (RMiESC) correction using seven principal components was performed on all spectra [45]. Afterwards, mean spectra for each group were averaged and generated using Origin 9.1 software with standard deviation (SD).

The integral intensities of selected IR bands of the nuclei and cytoplasm were calculated using the Opus software. Then, their box charts were constructed in the Origin Pro 9.4 software. The analysis of variance was carried out using the ANOVA statistical model, while determination of significance (*p*-values) was performed by Tukey’s test.

The pre-processed FTIR spectra of the nuclei and cytoplasm were analyzed using PCA provided by the Unscrambler X software in the regions of 3030–2820 cm^−1^ and 1780–1020 cm^−1^ by using the leave-out-one cross-validation approach and Nipal’s algorithm for PCA decomposition. Seven PCs were chosen for the initial decomposition and 20 iterations were performed for each PC.

Partial Least Squares Discriminant Analysis (PLS-DA) was performed for FTIR spectra of the cytoplasm and nuclei in the Unscrambler X software with similar pre-processing as PCA. Five models for discrimination of normal urothelial cells vs. carcinoma cell lines were built (N = 10 cells per cell line). The models included mean-centered seven factors. Then, PLS-DA prediction was performed on 10 other cells using loadings for two factors.

### 4.3. Raman Imaging, Spectral Pre-Processing and Analysis

RS images were collected with the use of Confocal Raman Imaging WITec Alpha 300, 63× water immersion objective (Zeiss, White Plains, NY, USA), 3 cm^−1^ spectral resolution and a step size of 1 μm spatial resolution with a 532 nm laser and the power only a little higher than the growth of a signal from the cell (it stops at some intensity), and integral time 0.5 s of one point. Recording of spectra facilitates the removal of cosmic rays and, additionally, all spectra were smoothed with a Savitzky–Golay filter (*k* = 3; *w* = 8). Then, background subtraction was performed (400–1800 and 2800–3000 cm^−1^ and a few points near 4000 cm^−1^) with *N* order shape 150.

Raman measurements of cells were recorded using a WITec confocal CRM alpha 300 Raman microscope. The spectrometer was equipped with an air-cooled solid state laser operating at 532 nm and a CCD detector (charge-coupled device), which was cooled down to −60 °C. The laser was coupled to the microscope via an optical fiber with a diameter of 50 µm. Raman measurements were performed using a 63× water immersion objective (Zeiss, NA = 1, White Plains, NY, USA). The spectra were recorded with a spectral resolution of 3 cm^−1^ and integration time of 0.6–0.7 s. The sampling density was 0.3–0.5 µm and it was adjusted to the size of the measured cells. The laser power measured before the objective was approximately 20 mW. Raman data analysis was performed using WITec software (WITec Plus, Ulm, Germany). Raman distribution images were obtained based on the integration of respective marker bands without spectral pre-processing. The Raman data were analyzed with the k-Means Cluster Analysis (KMCA) using the Manhattan distance algorithm. Cluster analysis was carried out after cosmic spike removal and background subtraction. The presented spectra were vector normalized in the range of 1500–400 cm^−1^. The integral intensity of selected bands was calculated using the Opus software. Then, ratios for box charts of bands were constructed in Origin Pro 9.4 software. The analysis of variance was carried out using the ANOVA statistical model, while determination of significance (*p*-values) was performed by Tukey’s test. Groups of 10–16 cells for each cell line were analyzed.

The CA k-means algorithm was applied to obtain nucleus, cytoplasm and cell signals. Spectral averages were loaded to OPUS cut at 350 cm^−1^ and normalized (vector normalization was separate in two regions: 1780–600 cm^−1^ and 3030–2800 cm^−1^ to present fingerprint and high numbers, particularly). Finally, all spectra for each group were averaged and generated using Origin Lab 2018 Pro software.

PCA, PLS-DA, and determination of integral intensities were performed in a similar manner as for FTIR spectra.

## 5. Conclusions

Our findings demonstrated that FTIR and Raman spectroscopy can be employed to distinguish between different bladder cancer cells of various malignancy. For the first time, we showed that both microscopic techniques revealed complementary information that could be useful in tracking metabolic changes. Chemism-induced spectroscopic features of the BC cells of various stages and invasiveness were specifically detected by both techniques. In comparison to other imaging techniques used in clinics, vibrational spectroscopy imaging is simple, label-free, sensitive and requires minimal sample preparation. Probing the cells with a spatial resolution of ca. 1 μm is enough to detect the main cellular compartments as well as storage organelles such as lipid bodies and graininess conventionally identified by specific staining. The advanced technological solutions to collect spectral data in a short time, e.g., quantum cascade lasers in IR microscopes and non-linear Raman techniques, shall also identify these cellular structures and support in vitro and clinical studies. Clustering of the cellular structures segregated nucleus from cytoplasm as well as graininess and lipid droplets were recognized in bladder cancer cells for the first time. We indicated herein the possible detection of S-S bridges that can be translated to a level of reduced glutathione and applied further to predict radiotherapy efficacy. In addition, it was proved that tumors derived from different stages present different metabolic changes that can be observed in FTIR and Raman spectra. Both deliver complementary information. Moreover, the same genetic lesions might have different biocomponent composition and, in consequence, different spectroscopic profiles. The same genetic “mother” of the T24 cell line gives T24a and T24p cells various metabolic profiles. A question arises about their malignancy and whether it is associated with contaminations from genetic information carriers from other cells, viruses or mitochondria. The FTIR and Raman images suggested epigenetic changes and the asymmetric division of nucleic acids. The results of our study may help to develop diagnostic methods and understand the steps of carcinogenesis. Furthermore, the proposed in vitro imaging technology can be useful in the search for novel and low-toxic therapeutics in personalized medicine. The findings of this study could provide a new analytical approach in a range of new imaging modalities for the first screening of cytological and histological samples. Currently, our group has been using a similar methodology on patient-derived samples; this will be the subject of future publications.

## Figures and Tables

**Table 1:** A summary of the cell line characteristics studied in this work.

Cell Line	HCV-29	T24a and T24p	RT4	HT-1376	J82
**Stage**	na	Ta	T1	T2	T3
**Grade**	na	high	low	high	high
**Type**	na	subtype dependent	luminal	mixed	basal
**Mutations**	na	HRAS, TERT, TP53	TERT, CDKN2A, TP53	FGFR3, PIK3CA, TERT, TP53	PIK3CA, TERT, TP53

Abbreviations: na—not applicable; Ta—papillary urothelial bladder cancer without invasion, T1—invasion of the sub-endothelial tissue, T2—invasion of the muscularis propria, T3—invasion of the tissues around the bladder but not other organs.

**Table 2:** A summary of biocomponents found in FTIR and Raman spectra of cellular compartments. ↑↑—high content; ↑—content present or similar but lower than in ↑↑; ↓—low content.

Biocomponent	HCV-29	Ta—T24a	Ta—T24p	T1-RT4	T2—HT-1376	T3—J82
	Nucleus					
Lipid content (FTIR)	↑	↑	↑	↑↑	↑	↑↑
Elongated acyl chains (FTIR)	↑	↑↑	↑↑	↓	↑↑	↑↑
Free fatty acids (FTIR)				↑		
Phospholipids (RS)	↑	↑	↑	↑	↑↑	↑↑
Cholesterol and its esters (RS)					↑↑	↑↑
Protein content (FTIR)				↑		
Tyr (RS)		↑	↑			
Uroplakin			↑			
Protein crosslinking (RS)	↑	↓	↑↑	↓	↑	↑
S-S bridge (RS)	↑			↑	↑↑	↑↑
DNA content (FTIR)				↑		
Glycogen (FTIR)				↑		
Carbohydrates (RS)	↑			↑↑	↑↑	↑↑
	Cytoplasm					
Lipid content (FTIR/RS)	↑	↑	↑	↑	↑	↑
Esterified fatty acids and cholesterol (FTIR)	↑	↑	↑	↑	↑	↑
Unsaturated fatty acids (RS)	↑↑	↑			↑↑	↑
Phospholipids (RS)	↑			↑↑	↑	↑
Protein content (FTIR)	↑	↑	↑	↑	↑	↑
Cytochromes (RS)	↑	↑↑	↑↑	↓	↑	↑
S-S bridge (RS)	↑			↑↑	↑↑	↑↑
Carbohydrates (RS)	↑			↑↑	↑	↑
	Graininess					
Lipid content (FTIR)		↑	↑	↑↑	↑↑	
Esterified fatty acids and cholesterol (FTIR)		↑	↑		↑	
Protein content (FTIR)		↑↑	↑↑	↑	↑	
Glycogen (FTIR)				↑		
Sugar moieties (FTIR; 1052 cm^−1^)		↑	↑↑		↑	
Nucleic acids (FTIR)		↑	↑	↑↑	↑	
RNA (FTIR)				↑		
	Lipid droplets					
Unsaturated fatty acids (RS)	↑		↑↑			
*Gauche* conformation of acyl chains (RS)	↑					
Phospholipids (RS)	↑					
Cholesterol and its esters (RS)	↑		↑↑			
S-S bridge (RS)	↑↑		↑			
Cytochromes (RS)	↑		↑↑			
Carbohydrates (RS)	↑↑		↑			

## Data Availability

The data presented in this study are available on request from the corresponding author. The data are not publicly available due to their massive file size.

## Supplementary Materials

The following are available online at https://www.mdpi.com/2072-6694/13/1/123/s1, Figure S1: Enlarged images of HE stained HE cell lines containing cellular structures similar to graininess (arrowheads): A—T24a, B—T24p, C—RT4, D—HT-1376; magnification 200×, Figure S2: HD-FTIR spectra of nuclei, cytoplasm and graininess spectra (N = 20 per cell line) derived from UHCA analysis of single cells., Figure S3: Averaged FTIR-transmission spectra of nuclei, cytoplasm and graininess and ATR-FTIR spectra of cell sediments, Figure S4: Changes in integral intensities of selected HD FTIR bands of cellular compartments, Figure S5: Changes in integral intensities of selected Raman bands of cellular compartments, Figure S6: 2-dimensional score plots of PCA analysis displayed in Figure 6, Figure S7: Partial Least Square Discrimination Analysis (PLS DA) of carcinoma and normal cells based on HD-FTIR spectra of nuclei, Figure S8: Partial Least Square Discrimination Analysis (PLS DA) of carcinoma and normal cells based on HD-FTIR spectra of cytoplasm, Figure S9: Partial Least Square Discrimination Analysis (PLS DA) of carcinoma and normal cells based on Raman spectra of nuclei, Figure S10: Partial Least Square Discrimination Analysis (PLS DA) of carcinoma and normal cells based on Raman spectra of cytoplasm, Table S1: FTIR band positions observed in infrared spectra of the urothelial lines with their assignment to vibrational modes and biomolecules, Table S2: RS band positions observed in Raman spectra of the urothelial lines with their assignment to vibrational modes and biomolecules, Table S3: PLS-DA parameters for discrimination of carcinoma (T24a, T24p, TR4, HT-1376, and J82) and normal urothelial cells (HCV-29) obtained for IR and Raman spectra of nuclei and cytoplasm.

## References

[1] Guo C.C., Czerniak B. (2019). Bladder cancer in the genomic era. Arch. Pathol. Lab. Med..

[2] Millis S.Z., Bryant D., Basu G., Bender R., Vranic S., Gatalica Z., Vogelzang N.J. (2015). Molecular profiling of infiltrating urothelial carcinoma of bladder and nonbladder origin. Clin. Genitourin. Cancer.

[3] Ward D.G., Gordon N.S., Boucher R.H., Pirrie S.J., Baxter L., Ott S., Silcock L., Whalley C.M., Stockton J.D., Beggs A.D. (2019). Targeted deep sequencing of urothelial bladder cancers and associated urinary DNA: A 23-gene panel with utility for non-invasive diagnosis and risk stratification. BJU Int..

[4] Garczyk S., Ortiz-Brüchle N., Schneider U., Lurje I., Guricova K., Gaisa N.T., Lorsy E., Lindemann-Docter K., Heidenreich A., Knüchel R. (2020). Next-generation sequencing reveals potential predictive biomarkers and targets of therapy for urothelial carcinoma in situ of the urinary bladder. Am. J. Pathol..

[5] Inamura K. (2018). Bladder cancer: New insights into its molecular pathology. Cancers.

[6] Zuiverloon T.C.M., De Jong F.C., Costello J.C., Theodorescu D. (2018). Systematic review: Characteristics and preclinical uses of bladder cancer cell lines. Bl. Cancer.

[7] Yoshida T., Sopko N.A., Kates M., Liu X., Joice G., McConkey D.J., Bivalacqua T.J. (2019). Impact of spheroid culture on molecular and functional characteristics of bladder cancer cell lines. Oncol. Lett..

[8] Lee S.H., Hu W., Matulay J.T., Silva M.V., Owczarek T.B., Kim K., Chua C.W., Barlow L.M.J., Kandoth C., Williams A.B. (2018). Tumor evolution and drug response in patient-derived organoid models of bladder cancer. Cell.

[9] Rodrigues D., Jerónimo C., Henrique R., Belo L., De Lourdes Bastos M., De Pinho P.G., Carvalho M. (2016). Biomarkers in bladder cancer: A metabolomic approach using in vitro and ex vivo model systems. Int. J. Cancer.

[10] Liu X., Cheng X., Liu X., He L., Zhang W., Wang Y., Sun W., Ji Z. (2018). Investigation of the urinary metabolic variations and the application in bladder cancer biomarker discovery. Int. J. Cancer.

[11] Diem M., Mazur A., Lenau K., Schubert J., Bird B., Miljković M., Krafft C., Popp J. (2013). Molecular pathology *via* IR and Raman spectral imaging. J. Biophoton..

[12] Wiercigroch E., Staniszewska-Slezak E., Szkaradek K., Wojcik T., Ozaki Y., Baranska M., Malek K. (2018). FT-IR spectroscopic imaging of endothelial cells response to tumor necrosis factor-α: To follow markers of inflammation using standard and high-magnification resolution. Anal. Chem..

[13] Harvey T.J., Hughes C., Ward A.D., Faria E.C., Henderson A., Clarke N.W., Brown M.D., Snook R.D., Gardner P. (2009). Classification of fixed urological cells using Raman tweezers. J. Biophoton..

[14] Kerr L.T., Adams A., O’Dea S., Domijan K., Cullen I., Hennelly B.M. (2014). Classification of bladder cancer cell lines using Raman spectroscopy: A comparison of excitation wavelength, sample substrate and statistical algorithms. Biophoton. Photon. Solut. Better Health Care IV.

[15] Kerr L.T., Lynn T.M., Cullen I.M., Daly P.J., Shah N., O’Dea S., Malkin A., Hennelly B.M. (2016). Methodologies for bladder cancer detection with Raman based urine cytology. Anal. Methods.

[16] Jen C.P., Huang C.T., Chen Y.S., Kuo C.T., Wang H.C. (2014). Diagnosis of human bladder cancer cells at different stages using multispectral imaging microscopy. IEEE J. Sel. Top. Quantum Electron..

[17] Canetta E., Mazilu M., De Luca A.C., Carruthers A.E., Dholakia K., Neilson S., Sargeant H., Briscoe T., Herrington C.S., Riches A.C. (2011). Modulated Raman spectroscopy for enhanced identification of bladder tumor cells in urine samples. J. Biomed. Opt..

[18] Gok S., Aydin O.Z., Sural Y.S., Zorlu F., Bayol U., Severcan F. (2016). Bladder cancer diagnosis from bladder wash by Fourier transform infrared spectroscopy as a novel test for tumor recurrence. J. Biophotonics.

[19] Yosef H.K., Krauß S.D., Lechtonen T., Jütte H., Tannapfel A., Käfferlein H.U., Brüning T., Roghmann F., Noldus J., Mosig A. (2017). Noninvasive diagnosis of high-grade urothelial carcinoma in urine by Raman spectral imaging. Anal. Chem..

[20] Hughes C., Iqbal-Wahid J., Brown M., Shanks J.H., Eustace A., Denley H., Hoskin P.J., West C., Clarke N.W., Gardner P. (2013). FTIR microspectroscopy of selected rare diverse sub-variants of carcinoma of the urinary bladder. J. Biophotonics.

[21] Shapiro A., Gofrit O.N., Pizov G., Cohen J.K., Maier J. (2011). Raman molecular imaging: A novel spectroscopic technique for diagnosis of bladder cancer in urine specimens. Eur. Urol..

[22] Crow P., Uff J.S., Farmer J.A., Wright M.P., Stone N. (2004). The use of Raman spectroscopy to identify and characterize transitional cell carcinoma in vitro. BJU Int..

[23] Stone N., Hart Prieto M.C., Crow P., Uff J., Ritchie A.W. (2007). The use of Raman spectroscopy to provide an estimation of the gross biochemistry associated with urological pathologies. Anal. Bioanal. Chem..

[24] Kessenbrock K., Plaks V., Werb Z. (2010). Matrix Metalloproteinases: Regulators of the tumor. Cell.

[25] Krauß S.D., Yosef H.K., Lechtonen T., Jütte H., Tannapfel A., Käfferlein H.U., Brüning T., Roghmann F., Noldus J., El-Mashtoly S.F. (2018). Integrating spatial, morphological, and textural information for improved cell type differentiation using Raman microscopy. J. Chemom..

[26] Bongiovanni G.A., Eynard A.R., Calderón R.O. (2005). Altered lipid profile and changes in uroplakin properties of rat urothelial plasma membrane with diets of different lipid composition. Mol. Cell. Biochem..

[27] Bohnert M. (2020). Tether Me, Tether me not—Dynamic organelle contact sites in metabolic rewiring. Dev. Cell.

[28] Sahu R.K., Argov S., Salman A., Huleihel M., Grossman N., Hammody Z., Kapelushnik J., Mordechai S. (2004). Characteristic absorbance of nucleic acids in the Mid-IR region as possible common biomarkers for diagnosis of malignancy. Technol. Cancer Res. Treat..

[29] Staniszewska E., Malek K., Baranska M. (2014). Rapid approach to analyze biochemical variation in rat organs by ATR FTIR spectroscopy. Spectrochim. Acta Part A Mol. Biomol. Spectrosc..

[30] Banyay M., Sarkar M., Gräslund A. (2003). A library of IR bands of nucleic acids in solution. Biophys. Chem..

[31] Whelan D.R., Bambery K.R., Heraud P., Tobin M.J., Diem M., McNaughton D., Wood B.R. (2011). Monitoring the reversible B to A-like transition of DNA in eukaryotic cells using Fourier transform infrared spectroscopy. Nucleic Acids Res..

[32] Brandenburg K., Seydel U. (1998). Infrared spectroscopy of glycolipids. Chem. Phys. Lipids.

[33] Bik E., Dorosz A., Mateuszuk L., Baranska M., Majzner K. (2020). Fixed versus live endothelial cells: The effect of glutaraldehyde fixation manifested by characteristic bands on the Raman spectra of cells. Spectrochim. Acta Part A Mol. Biomol. Spectrosc..

[34] Majzner K., Chlopicki S., Baranska M. (2016). Lipid droplets formation in human endothelial cells in response to polyunsaturated fatty acids and 1-methyl-nicotinamide (MNA); confocal Raman imaging and fluorescence microscopy studies. J. Biophotonics.

[35] Prescott B., Steinmetz W., Thomas G.J. (1984). Characterization of DNA structures by laser Raman spectroscopy. Biopolymers.

[36] Brazhe N.A., Treiman M., Brazhe A.R., Find N.L., Maksimov G.V., Sosnovtseva O.V. (2012). Mapping of Redox State of Mitochondrial Cytochromes in Live Cardiomyocytes Using Raman Microspectroscopy. PLoS ONE.

[37] Nawaz H., Garcia A., Meade A.D., Lyng F.M., Byrne H.J. (2013). Raman micro spectroscopy study of the interaction of vincristine with A549 cells supported by expression analysis of bcl-2 protein. Analyst.

[38] Schulze A., Harris A.L. (2012). How cancer metabolism is tuned for proliferation and vulnerable to disruption. Nature.

[39] Massari F., Ciccarese C., Santoni M., Iacovelli R., Mazzucchelli R., Piva F., Scarpelli M., Berardi R., Tortora G., Lopez-Beltran A. (2016). Metabolic phenotype of bladder cancer. Cancer Treat. Rev..

[40] Gerresheim G.K., Roeb E., Michel A.M., Niepmann M. (2019). Oxidative phosphorylation, reminiscent of the Warburg effect in cancer cells. Cells.

[41] Mulcahy Levy J.M., Thorburn A. (2020). Autophagy in cancer: Moving from understanding mechanism to improving therapy responses in patients. Cell Death Differ..

[42] Yue S., Li J., Lee S.Y., Lee H.J., Shao T., Song B., Cheng L., Masterson T.A., Liu X., Ratliff T.L. (2014). Cholesteryl ester accumulation induced by PTEN loss and PI3K/AKT activation underlies human prostate cancer aggressiveness. Cell Metab..

[43] Su Y., Zhao B., Zhou L., Zhang Z., Shen Y., Lv H., AlQudsy L.H.H., Shang P. (2020). Ferroptosis, a novel pharmacological mechanism of anti-cancer drugs. Cancer Lett..

[44] Lasch P. CytospecTM. A Matlab Based Application for Infrared Imaging. http://www.cytospec.com.

[45] Bassan P., Kohler A., Martens H., Lee J., Byrne H.J., Dumas P., Gazi E., Brown M., Clarke N., Gardner P. (2010). Resonant Mie Scattering (RMieS) correction of infrared spectra from highly scattering biological samples. Analyst.

