# Supplementary material for: In Vitro Spectroscopy-Based Profiling of Urothelial Carcinoma: A Fourier Transform Infrared and Raman Imaging Study
Source: Cancers (Basel). 2021 Jan 2;13(1):123. doi: 10.3390/cancers13010123 (PMC7796146; doi:10.3390/cancers13010123)
Supplement: Supplementary file 1 [file cancers-13-00123-s001.pdf]

Supplementary Materials

In vitro spectroscopy-based profiling of urothelial carcinoma: A Fourier transform Infrared and Raman imaging study

Monika Kujdowicz[1,2], Wojciech Placha[3], Brygida Mech[2], Karolina Chrabaszcz[2], Krzysztof Okoń[1] and Kamilla Malek [2,*]

1 Department of Pathology, Faculty of Medicine, Jagiellonian University Medical College, Krakow, Grzegorzecka 16, 31-531, Poland

2 Faculty of Chemistry, Jagiellonian University in Krakow, Krakow, Gronostajowa 2, 30-387, Poland

3 Chair of Medical Biochemistry, Faculty of Medicine, Jagiellonian University Medical College, Krakow, Kopernika 7, 31-034, Poland

* Correspondence: kamilla.malek@uj.edu.pl

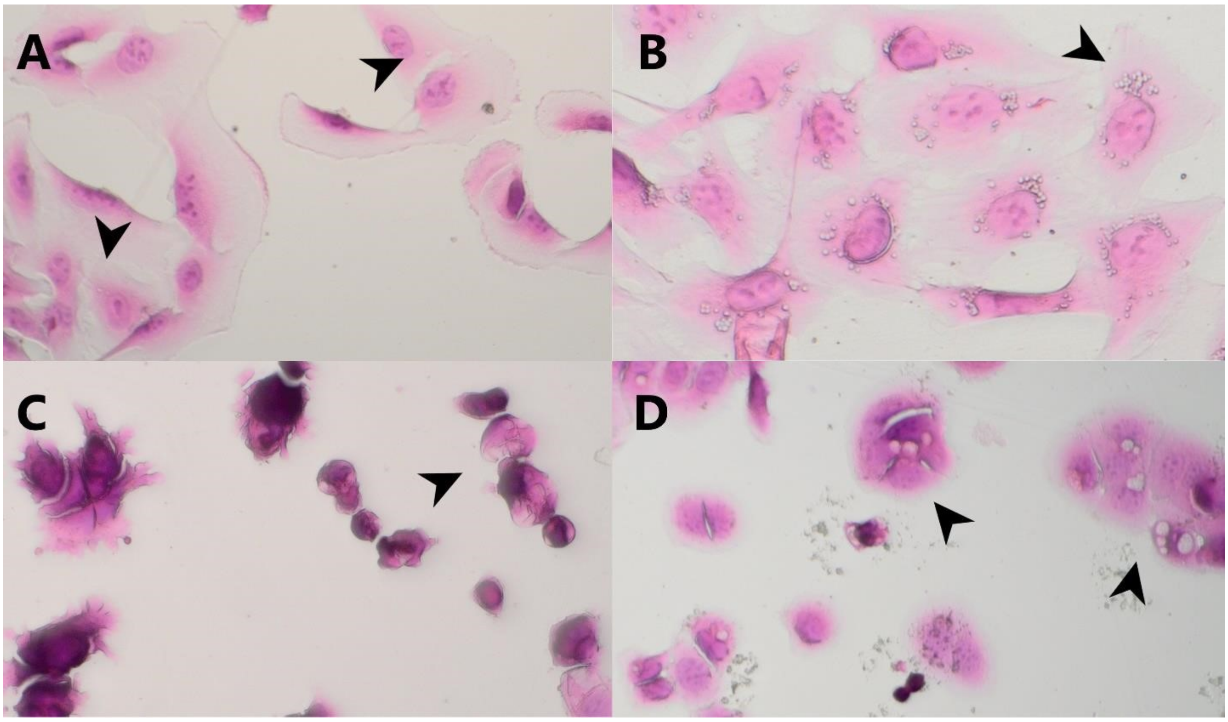

Figure S1. Enlarged images of HE stained HE cell lines containing cellular structures similar to graininess (arrowheads): A- T24a, B – T24p, C – RT4, D – HT-1376; magnification 200x.

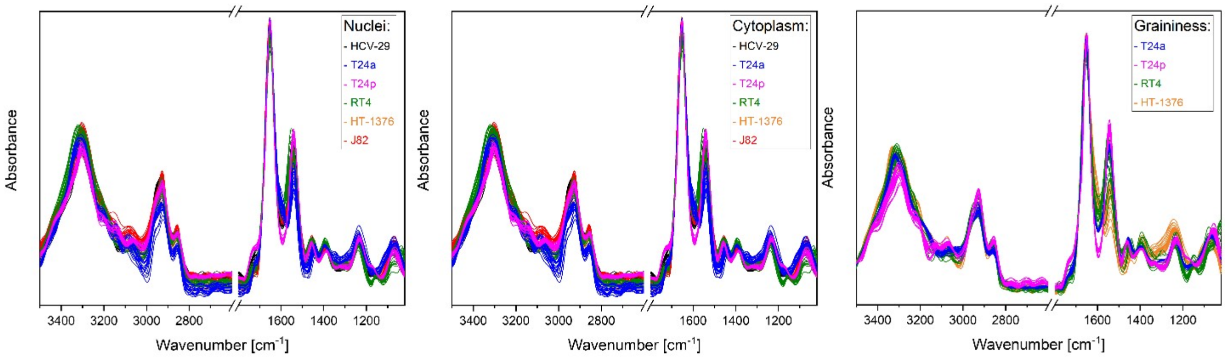

Figure S2. HD-FTIR spectra of nuclei, cytoplasm and graininess spectra (N = 20 per cell line) derived from UHCA analysis of single cells.

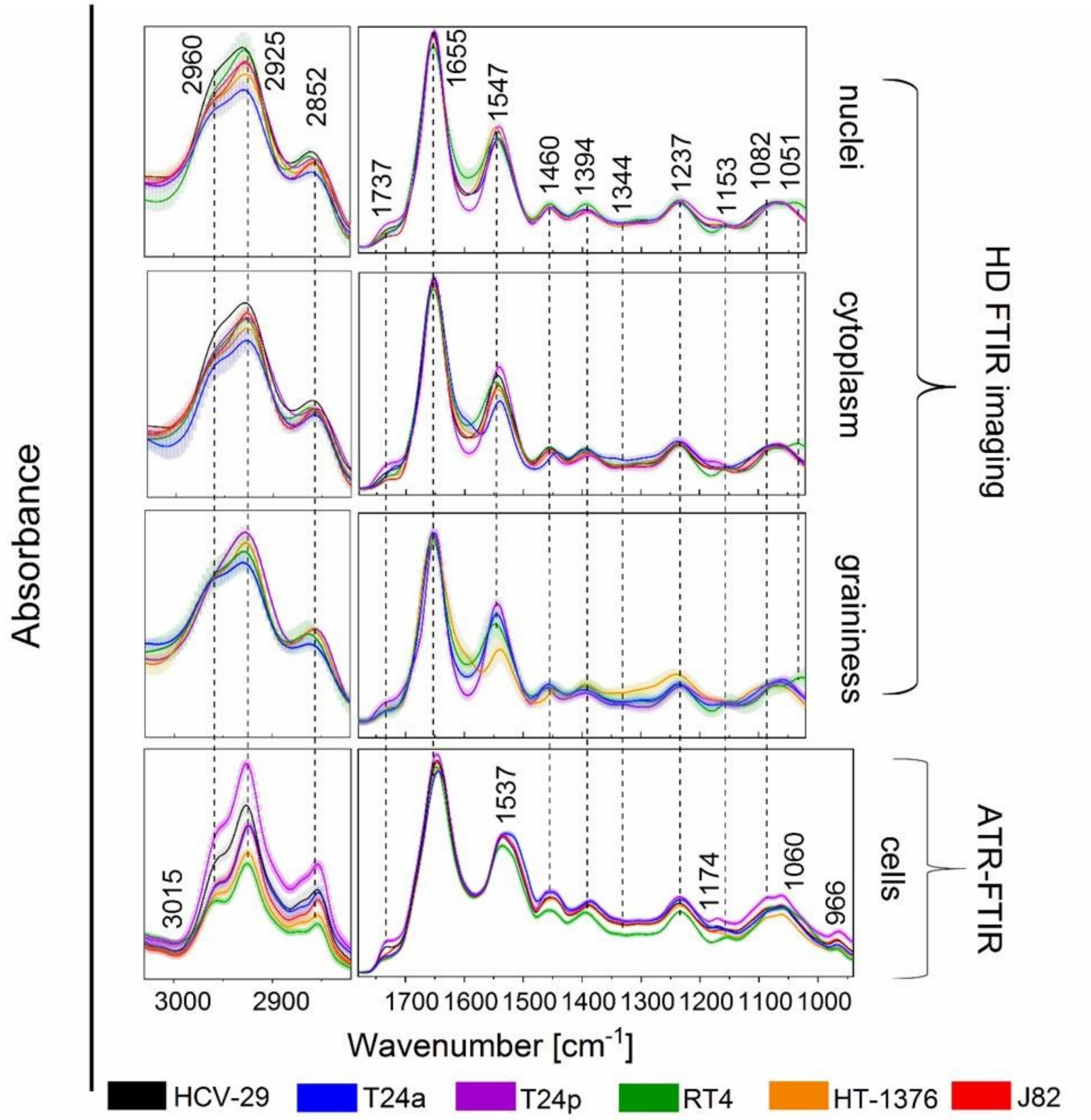

Figure S3. Averaged FTIR-transmission spectra of nuclei, cytoplasm and graininess and ATR-FTIR spectra of cell sediments. Spectra of cellular compartments were extracted from UHCA analysis of HD FTIR images. Gray shading denotes standard deviation (±SD).

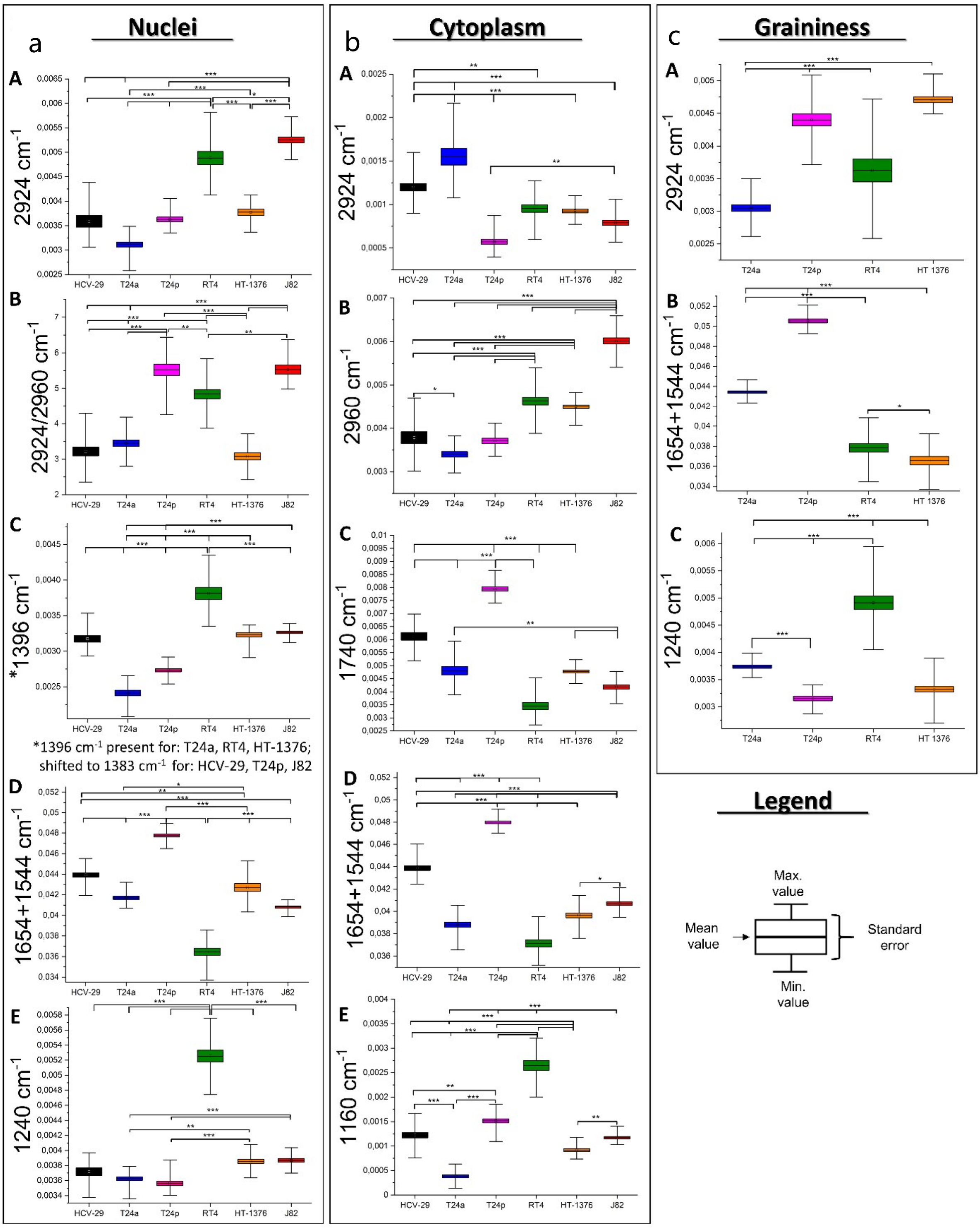

Figure S4. Changes in integral intensities of selected HD FTIR bands of cellular compartments.

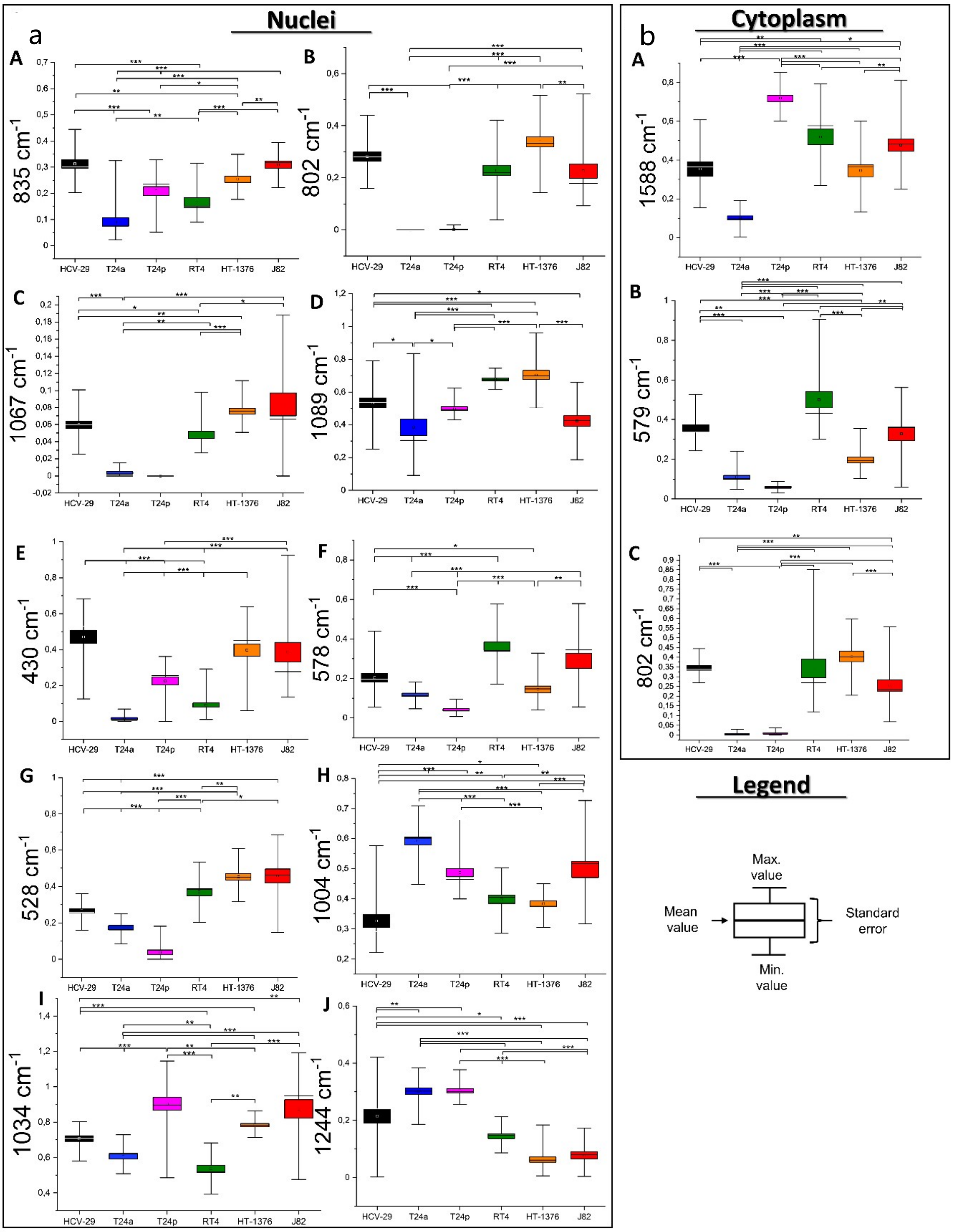

Figure S5. Changes in integral intensities of selected Raman bands of cellular compartments.

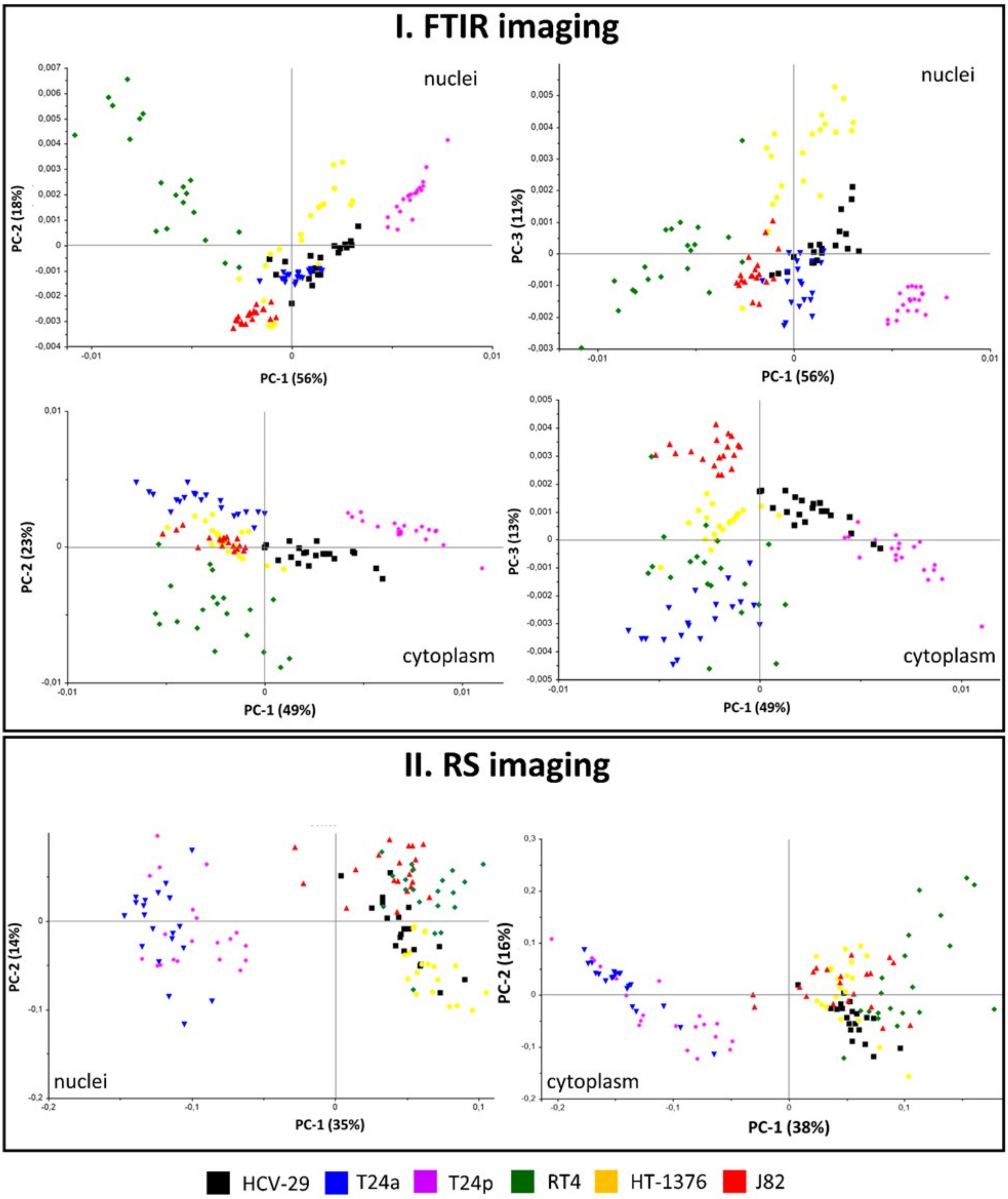

Figure S6. 2-dimensional score plots of PCA analysis displayed in Figure 6.

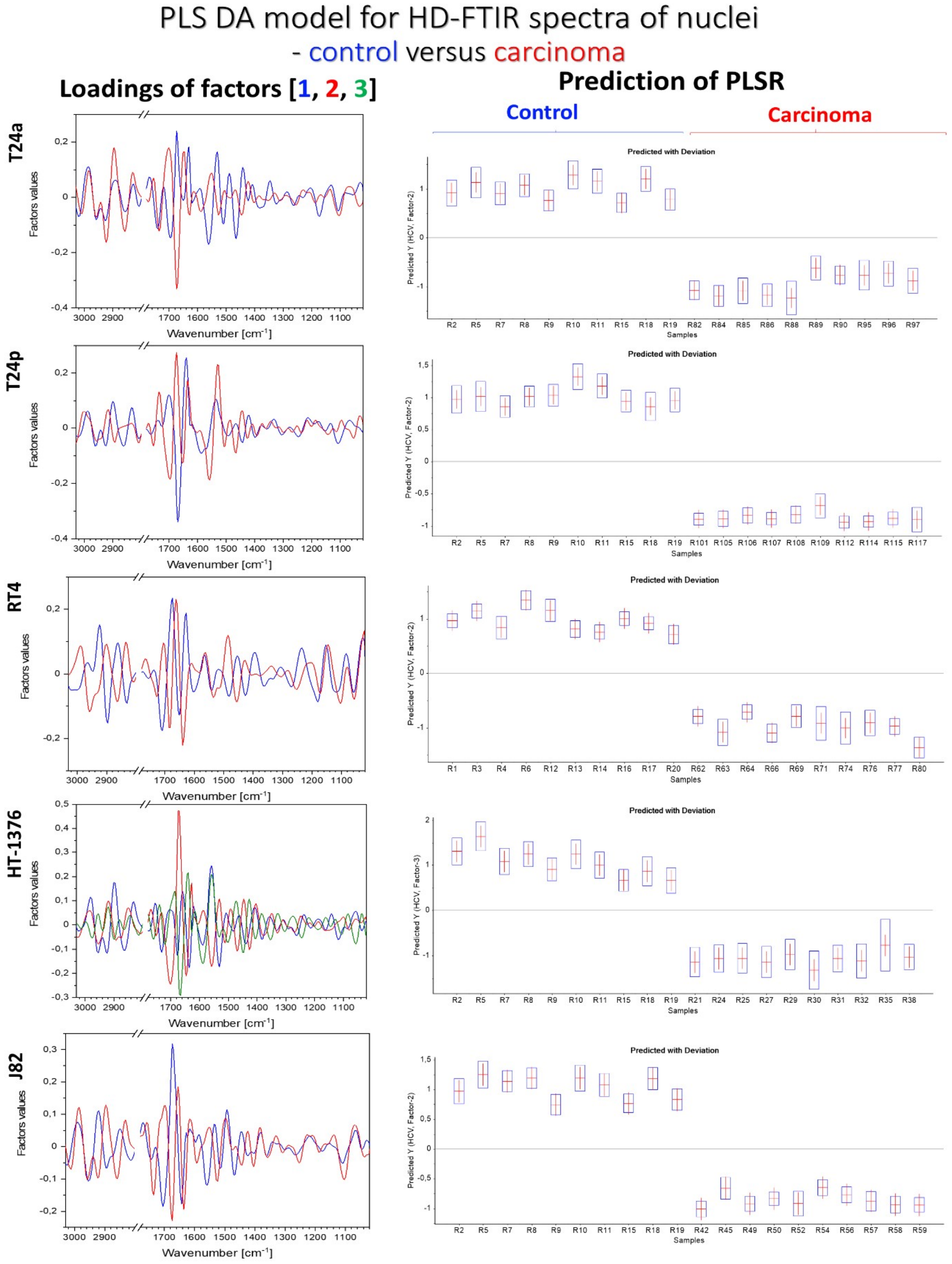

Figure S7. Partial Least Square Discrimination Analysis (PLS DA) of carcinoma and normal cells based on HD-FTIR spectra of nuclei.

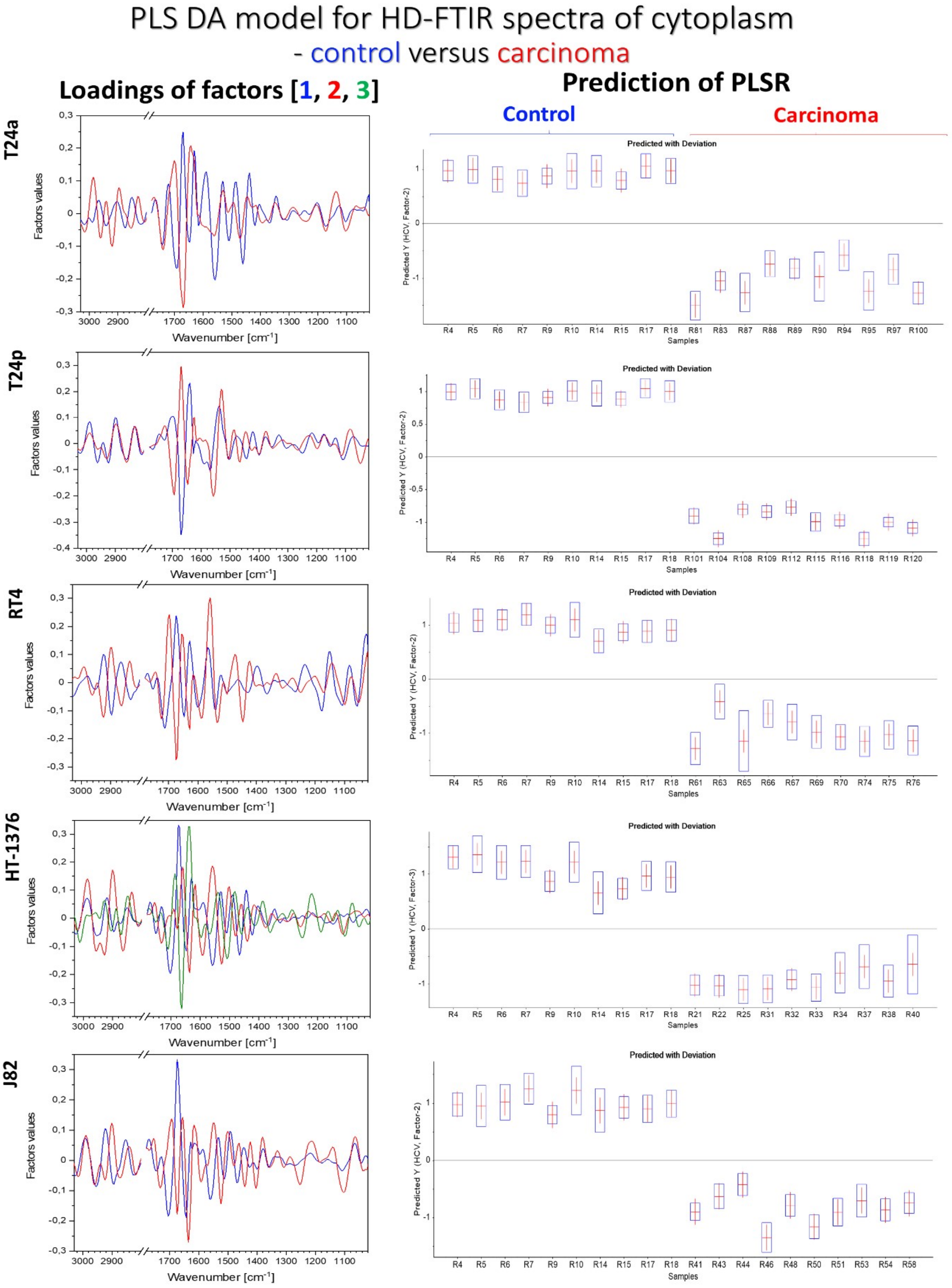

Figure S8. Partial Least Square Discrimination Analysis (PLS DA) of carcinoma and normal cells based on HD-FTIR spectra of cytoplasm.

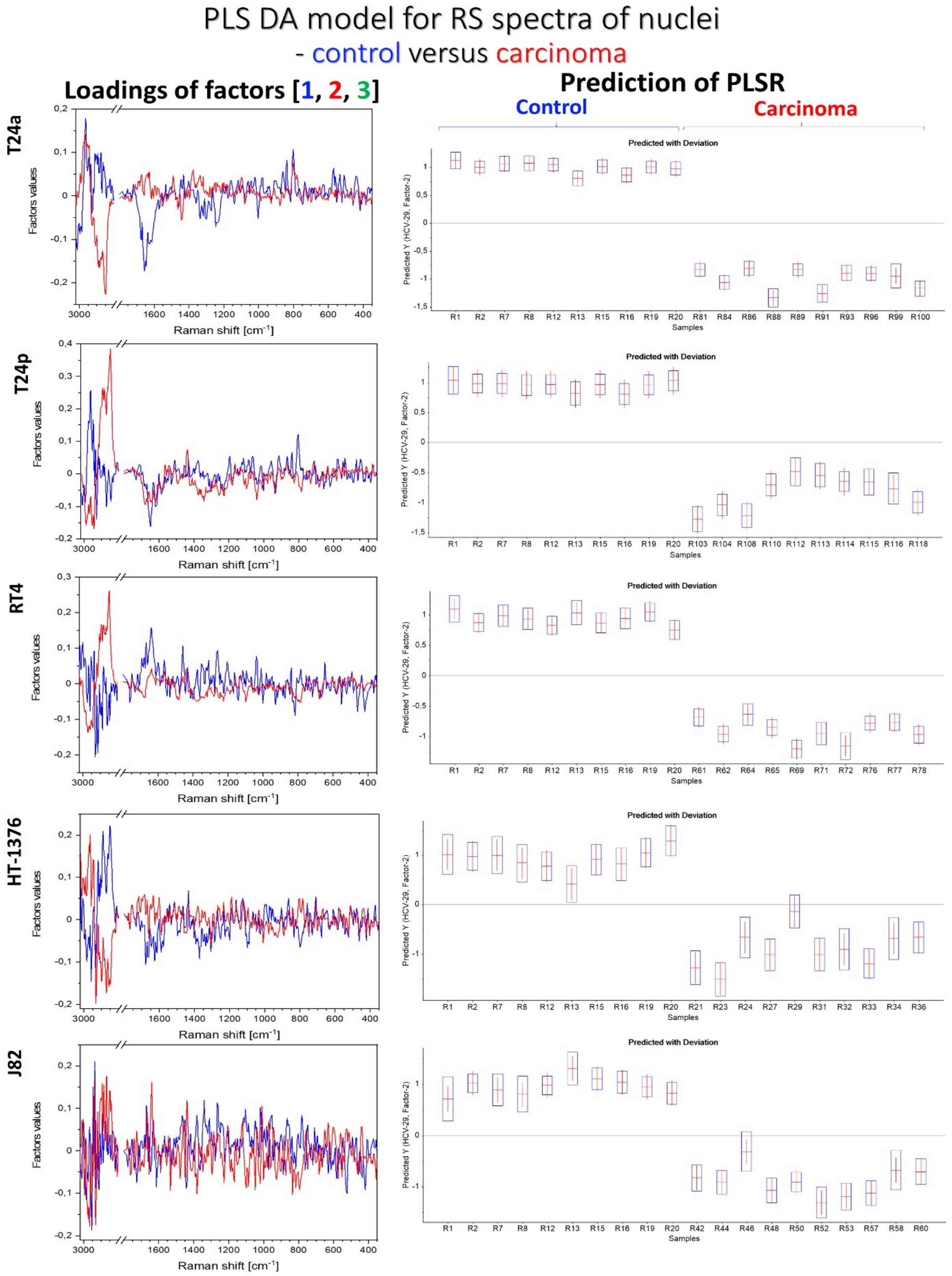

Figure S9. Partial Least Square Discrimination Analysis (PLS DA) of carcinoma and normal cells based on Raman spectra of nuclei.

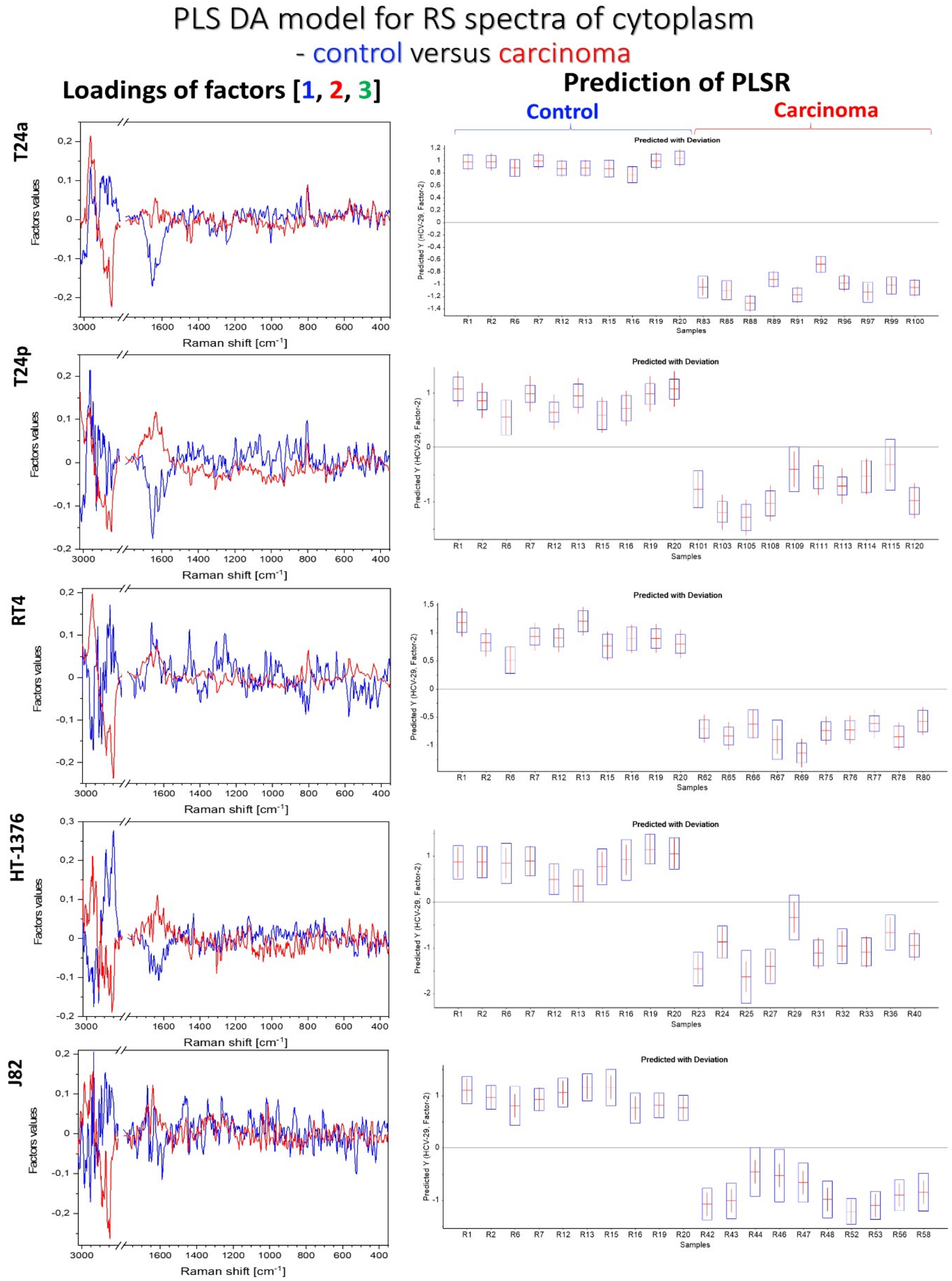

Figure S10. Partial Least Square Discrimination Analysis (PLS DA) of carcinoma and normal cells based on Raman spectra of cytoplasm.

Table S1. FTIR band positions observed in infrared spectra of the urothelial lines with their assignment to vibrational modes and biomolecules [1 – 5].

Position [cm^{-1}]	Assignment to biomolecules and vibrational modes
**922**	Carbohydrates; ν(C–C), ν(C–O)
**966**	DNA; ν(C-C)
**996**	RNA; Ring stretch and deformation of uracil
**1022-1041**	Carbohydrates, glycogen; ν(C–O)
**1054**	Carbohydrates, glycoproteins, glycolipids; ν(C–O)<br>Nucleic acids; backbone ν(C–O)<br>Cholesterol; ν(C–O)
**1081**	Nucleic acids; $\nu_s(PO_2^-)$<br>Phospholipids; $\nu_s(PO_2^-)$<br>Glycogen; ν(C-C)
**1125**	Ribose (RNA); ν(C-O)<br>Polysaccharides; ν(CC-OC)
**1153**	Glycogen; ν_{as}(CO-O-C)<br>Polysaccharides; ν_{as}(CO-O-C)
**1160**	Fatty acids and cholesterol esters; ν(C-O)
**1240**	Nucleic acids, phospholipids, phosphoproteins; $\nu_{as}(PO_2^-)$
**1282**	Proteins (amide III); ν(C-N) and ν(C-C)
**1309**	Proteins (amide III); ν(C-N) and ν(C-C)
**1344**	Phospholipids, fatty acids, triglycerides; δ(CH_2)<br>Amino acid side chains; δ(CH_2)
**1396**	Free fatty acids; $\nu_s(COO^-)$<br>Free amino acids; $\nu_s(COO^-)$
**1445**	Lipids; δ(CH_2, CH_3)
**1461**	Proteins; δ(CH_2, CH_3)<br>C (DNA); δ(NH), ν(CC)
**1518**	Tyr (proteins); ν(CC) of the Tyr ring<br>C (methylated DNA); in-plane vibrations of the ring
**1544**	Proteins (amide II); δ(N-H) and ν(C-N)
**1586**	G (DNA); ν(C=C-C) of ring

**1595**	Free amino acids; $\nu_{as}(COO^{-})$
**1648-1654**	α-Helices in proteins (amide I); ν(C=O) and δ(N-H)
**1682**	β-turns in proteins (amide I); ν(C=O) and δ(N-H)<br>G (DNA); ν(C=O) and ν(C=C)
**Position [cm^{-1}]**	**Assignment to biomolecules and vibrational modes**
**1718**	Fatty acids; ν(C=O)<br>Base pair (B-DNA); ν(C=O)
**1735**	Cholesterol esters; ν_{ester}(C=O)
**1740**	Triacylglycerols; ν_{ester}(C=O)
**2850**	Long chain fatty acids; $\nu_{s}(CH_2)$
**2875**	Proteins, lipids, nucleic acids; $\nu_{s}(CH_3)$
**2895**	Terminal CH_3 group in acyl chains (lipids); ν(CH)
**2924**	Lipids and proteins; $\nu_{as}(CH_2)$
**2960**	Proteins, lipids; $\nu_{as}(CH_3)$
**3014**	Unsaturated fatty acids; ν(=C-H)

ν – stretching mode, as – asymmetric, s – symmetric; δ – in-plane deformations; G – guanine; C – cytosine; Tyr – tyrosine.

Table S2. RS band positions observed in Raman spectra of the urothelial lines with their assignment to vibrational modes and biomolecules [6 – 11].

Position [cm^{-1}]	Assignment to biomolecules and vibrational modes
**430**	Cholesterol, cholesterol esters
**528**	*ggt* conformation of S-S bonds in proteins; ν(S-S)
**579**	Carbohydrates, Trp; δ(C-C=O)
**609**	Cholesterol, cholesterol esters; ν(steroid ring)
**647**	Tyr
**701**	Cholesterol, cholesterol esters; ν(steroid ring)
**722**	A (nucleic acids); ring breathing<br>Phospholipids<br>Proteins; ν(C-S)
**751**	Cyt. c, c_1 and b; ν_{15}, ν_s(pyr deform)sym
**785**	DNA; ν_{as}(OPO)<br>U, T, C (nucleic acids)
**802**	Phospholipids; ν(OPO)<br>Nucleic acids; δ(CH-CHO)
**835**	Tyr (proteins); ring breathing<br>DNA; ν_s(OPO)<br>DNA B<br>Glucose; ν(C-C) and ν(C-O-C-O)
**860**	Tyr (proteins); ν_s(C-C-N^+)
**Position [cm^{-1}]**	**Assignment to biomolecules and vibrational modes**
**893**	Trp (proteins); ν(C-C) and ν(C-N)
**930**	Cholesterol esters (lipid droplets); ν(C-C)
**935**	Protein (keratin like structure in uroplakin); ν(C-C)
**962**	Carbohydrates; ν(C-O)<br>Nucleic acids; phosphodiester chain
**1004**	Phe (proteins); ring breathing
**1034**	Proteins crosslinking; δ(C-H) and δ(C-N)
**1067**	Lipids (*gauche* in acyl backbone); ν(C-C)

**1089**	Phospholipids; ν(PO_2^-)<br>Lipids (*trans* in acyl backbone); ν(C-C)
**1129**	Lipids (*trans* in acyl backbone); ν(C-C)<br>Cyt; ν(C-N)<br>Proteins; ν(C-O)<br>Carbohydrates; ν(C-O)
**1179**	Tyr, Phe (proteins); δ(C-H)<br>C, G (nucleic acids); ν(C-C)
**1209**	Tyr, Phe, Trp, Hyp (proteins); τ(CH_2)
**1244**	Amide III (likely uroplakin); ν(C-N) and δ(N-H)
**1268**	Amide III; ν(C-N) and δ(N-H)<br>Lipids; δ(=CH)
**1280**	Lipids; δ(CH_2)
**1307**	Lipids; τCH_2-CH_3
**1344**	A (nucleic acids); δ(CH)<br>Proteins; δ(CH)<br>Carbohydrates; δ(CH)<br>Reduced cyt. b
**1375**	A (nucleic acids); δ(CH_3)
**1447**	Proteins, lipids; δ(CH_2), δ(CH_3)
**1588**	Reduced cyt. c and b
**1660**	Proteins (amide I); ν(C=O) and δ(N-H)<br>Unsaturated fatty acids; ν(C=C)
**1725**	Cholesterol esters; ν_{ester}(C=O)
**1737**	Triacylglycerols, ν_{ester}(C=O)
**2852**	Long chain fatty acids; ν_s(CH_2)
**2875**	Lipids, proteins; ν(C-H)-CH_2
**2893**	Lipids, proteins; ν_s(-C-H)-CH_3
**Position [cm^{-1}]**	**Assignment to biomolecules and vibrational modes**
**2935**	Lipids, proteins; ν(C-H)
**2962**	Nucleic acids, lipids; ν_{as}(CH_3), ν_{as}CH(-CH_2)

**3015**	Unsaturated fatty acids; ν(=C-H)

ν – stretching mode, as – asymmetric, s – symmetric; δ – in-plane deformations; τ – twisting; cyt – cytochromes; A – adenine; C – cytosine, G – guanine; U – uracil; T – thymine; Tyr – tyrosine; Phe – phenylalanine; Trp – tryptophan; Hyp – proline.

Table S3. PLS-DA parameters for discrimination of carcinoma (T24a, T24p, TR4, HT-1376, and J82) and normal urothelial cells (HCV-29) obtained for IR and Raman spectra of nuclei and cytoplasm.

**Cancer cell line**	**T24a**	**T24p**	**RT4**	**HT-1376**	**J82**
**PLS parameters**	**RMSE / R^2**	**RMSE / R^2**	**RMSE / R^2**	**RMSE / R^2**	**RMSE / R^2**
**HD-FTIR spectra of nuclei**					
Calibration	0.23 / 0.95	0.17 / 0.97	0.19 / 0.96	0.30 / 0.91	0.17 / 0.97
Validation	0.33 / 0.90	0.23 / 0.95	0.23 / 0.95	0.46 / 0.81	0.24 / 0.95
Prediction	0.21 / 0.96	0.14 / 0.98	0.19 / 0.96	0.24 / 0.94	0.19 / 0.97
**HD-FTIR spectra of cytoplasm**					
Calibration	0.22 / 0.95	0.13 / 0.98	0.27 / 0.93	0.16 / 0.97	0.20 / 0.96
Validation	0.28 / 0.93	0.16 / 0.98	0.35 / 0.89	0.33 / 0.90	0.27 / 0.93
Prediction	0.22 / 0.95	0.13 / 0.98	0.21 / 0.96	0.21 / 0.96	0.23 / 0.95
**RS spectra of nuclei**					
Calibration	0.12 / 0.99	0.19 / 0.96	0.15 / 0.98	0.29 / 0.91	0.16 / 0.97
Validation	0.16 / 0.98	0.25 / 0.94	0.22 / 0.96	0.43 / 0.83	0.30 / 0.92
Prediction	0.14 / 0.98	0.23 / 0.95	0.17 / 0.97	0.32 / 0.90	0.24 / 0.94
**RS spectra of cytoplasm**					
Calibration	0.10 / 0.99	0.20 / 0.96	0.20 / 0.96	0.34 / 0.88	0.21 / 0.95
Validation	0.15 / 0.98	0.26 / 0.94	0.28 / 0.93	0.47 / 0.80	0.37 / 0.87
Prediction	0.14 / 0.98	0.89 / 0.89	0.25 / 0.94	0.33 / 0.89	0.22 / 0.95

References

1. Sahu, R.K.; Argov, S.; Salman, A.; Huleihel, M.; Grossman, N.; Hammody, Z.; Kapelushnik, J.; Mordechai, S Mordechai. Characteristic absorbance of nucleic acids in the Mid-IR region as possible common biomarkers for diagnosis of malignancy. *Technol. Cancer Res. Treat.* **2004**, *3*, 629–638.
2. Staniszewska, E.; Malek, K.; Baranska, M. Rapid approach to analyze biochemical variation in rat organs by ATR FTIR spectroscopy. *Spectrochim. Acta - Part A Mol. Biomol. Spectrosc.* **2014**, *118*, 981–986.
3. Wiercigroch, E.; Staniszewska-Slezak, E.; Szkaradek, K.; Wojcik, T.; Ozaki, Y.; Baranska, M.; Malek, K. FT-IR Spectroscopic Imaging of Endothelial Cells Response to Tumor Necrosis Factor-α: To Follow Markers of Inflammation Using Standard and High-Magnification Resolution. *Anal. Chem.* **2018**, *90*, 3727–3736.
4. Banyay, M.; Sarkar, M.; Gräslund, A. A library of IR bands of nucleic acids in solution. *Biophys. Chem.* **2003**, *104*, 477–488.
5. Whelan, D.R.; Bambery, K.R.; Heraud, P.; Tobin, M.J.; Diem, M.; McNaughton, D.; Wood, B.R. Monitoring the reversible B to A-like transition of DNA in eukaryotic cells using Fourier transform infrared spectroscopy. *Nucleic Acids Res.* **2011**, *39*, 5439–5448.
6. Bik, E.; Dorosz, A.; Mateuszuk, L.; Baranska, M.; Majzner, K. Fixed versus live endothelial cells: The effect of glutaraldehyde fixation manifested by characteristic bands on the Raman spectra of cells. *Spectrochim. Acta - Part A Mol. Biomol. Spectrosc.* **2020**, *240*, 118460.
7. Majzner, K.; Chlopicki, S.; Baranska, M. Lipid droplets formation in human endothelial cells in response to polyunsaturated fatty acids and 1-methyl-nicotinamide (MNA); confocal Raman imaging and fluorescence microscopy studies. *J. Biophotonics* **2016**, 9, 396–405.
8. Prescott, B.; Steinmetz W.; Thomas, G. J. Characterization of DNA structures by laser Raman spectroscopy. *Biopolymers* **1984**, *23*, 235–256.
9. Brazhe, N.A.; Treiman, M.; Brazhe, A. R.; Find, N.L.; Maksimov, G.V.; Sosnovtseva, O.V. Mapping of Redox State of Mitochondrial Cytochromes in Live Cardiomyocytes Using Raman Microspectroscopy. *PLoS One* **2012**, *7*, 1–8.
10. Harvey, T.J.; Hughes, C.; Ward, A.D.; Faria, E.C.; Henderson, A.; Clarke, N.W.; Brown, M.D.; Snook, R.D.; Gardner P. Classification of fixed urological cells using Raman tweezers. *J. Biophotonics*. **2009**, 2, 47–69.
11. Jen, C.P.; Huang, C. Te.; Chen, Y.S.; Kuo C.T.; Wang, H. C. Diagnosis of human bladder cancer cells at different stages using multispectral imaging microscopy. *IEEE J. Sel. Top. Quantum Electron.* **2014**, *20*, 6800808.
